# Obtaining Arbitrary Prescribed Mean Field Dynamics for Recurrently Coupled Networks of Type-I Spiking Neurons with Analytically Determined Weights

**DOI:** 10.3389/fncom.2016.00015

**Published:** 2016-02-29

**Authors:** Wilten Nicola, Bryan Tripp, Matthew Scott

**Affiliations:** ^1^Department of Applied Mathematics, University of WaterlooWaterloo, ON, Canada; ^2^Department of Systems Design Engineering, University of WaterlooWaterloo, ON, Canada; ^3^Center for Theoretical Neuroscience, University of WaterlooWaterloo, ON, Canada

**Keywords:** mean field analysis, neural engineering framework, neuronal heterogeneity, integrate-and-fire neurons, recurrently coupled networks, synaptic weights

## Abstract

A fundamental question in computational neuroscience is how to connect a network of spiking neurons to produce desired macroscopic or mean field dynamics. One possible approach is through the Neural Engineering Framework (NEF). The NEF approach requires quantities called decoders which are solved through an optimization problem requiring large matrix inversion. Here, we show how a decoder can be obtained analytically for type I and certain type II firing rates as a function of the heterogeneity of its associated neuron. These decoders generate approximants for functions that converge to the desired function in mean-squared error like 1/N, where N is the number of neurons in the network. We refer to these decoders as scale-invariant decoders due to their structure. These decoders generate weights for a network of neurons through the NEF formula for weights. These weights force the spiking network to have arbitrary and prescribed mean field dynamics. The weights generated with scale-invariant decoders all lie on low dimensional hypersurfaces asymptotically. We demonstrate the applicability of these scale-invariant decoders and weight surfaces by constructing networks of spiking theta neurons that replicate the dynamics of various well known dynamical systems such as the neural integrator, Van der Pol system and the Lorenz system. As these decoders are analytically determined and non-unique, the weights are also analytically determined and non-unique. We discuss the implications for measured weights of neuronal networks.

## 1. Introduction

There are many spiking models that exist in the literature that can be fit to reproduce the membrane potential and the firing rates of real neurons. Examples include the leaky integrate and fire neuron, the Izhikevich model (Izhikevich, 2003, 2007), the theta model (Ermentrout and Kopell, 1986), the quartic integrate and fire model (Touboul, 2008) and the adaptive exponential integrate and fire model (Brette and Gerstner, 2005; Naud et al., 2008). When these models are coupled together to form networks, one can predict the the macroscopic or mean field behavior of a network of these neurons via a suitably derived mean field system (Nicola and Campbell, 2013a,b; Nesse et al., 2008). This can even be done when one considers the effects of heterogeneity in the neurons (Nicola and Campbell, 2013b).

While the mean field system for networks of neurons with prescribed sources of heterogeneity is important for predicting the behavior of the network of equal importance is the inverse problem: given a particular macroscopic behavior or mean field system, what distributions of heterogeneity, either in the neuronal parameters themselves, or the synaptic weights are required to produce said behavior?

One possible numerical solution to the inverse problem is through the Neural Engineering Framework (NEF) (Eliasmith and Anderson, 2004). In the NEF approach, one can specify the macroscopic dynamics or mean field for a spiking neuronal network. Given a network of neurons with a source of heterogeneity, one can find a set of optimal linear weights, referred to as linear decoders, for their firing rates in such a way that the weighted linear sum of the firing rates optimally approximates any function of choice. This allows for specifying the network connectivity in such a way as to obtain arbitrary dynamics from the network(s) of neurons (Eliasmith, 2005). For example, the NEF has been used to develop a wide variety of models, including the most behaviorally sophisticated spiking neural model to date (Eliasmith et al., 2012) as well as more specialized models of path integration (Conklin and Eliasmith, 2005), working memory (Singh and Eliasmith, 2006), visual attention (Bobier et al., 2014), motor control (DeWolf and Eliasmith, 2011), various cognitive functions (Bekolay et al., 2014; Rasmussen and Eliasmith, 2014), and many others.

However, the optimality requirement in the linear decoders introduces complications in the NEF approach. The optimal decoders are computed via least-squares optimization which is a computationally-intensive process; and yet very little information about the network can be determined once the optimal decoders have been obtained. Additionally, one cannot determine how the distribution of heterogeneity in the tuning curves of the neurons is related to the other distributions across the network, such as the distribution of connection weights.

Here, we will show that if one loosens the optimality requirement in the linear decoders, it is possible to obtain linear decoders that converge to any function of choice in the large network limit. Due to their form, we will refer to these decoders as scale-invariant linear decoders. These scale-invariant decoders have several advantages over optimal decoders, at the primary cost of a slower convergence rate in network size. However, using any gradient descent algorithm that does not directly compute the Hessian, one can decrease the error of the scale-invariant decoders very rapidly with very few iterations for any finite network size.

In Section 2.1, we will quickly introduce the NEF. A more thorough introduction can be found in (Eliasmith and Anderson, 2004). This will be followed by Section 2.2 where we will demonstrate that as the networks become arbitrarily large, the optimal decoders tend to an asymptotic limit. This will be our motivation in defining a scale-invariant decoder. In Section 2 we will determine what this asymptotic limit is for the scalar case and for multivariable functions in Section 2.4. In Section 3 we will demonstrate how the decoders can yield the weights to couple neurons together and simulate spiking networks with the specified dynamics by using these weights.

## 2. Methods: determining the decoder surface

### 2.1. The neural engineering framework

Suppose we knew the firing rate of a class of neurons, *f*(*I*) as a function of the input current *I*. Then we can take any input variable *x* and linearly transform it into a current via *I* = α*x* + β. If we allow α and β to be drawn from a random distribution, then we can generate a network of neurons with firing rates *f*(α_*i*_*x* + β_*i*_) where α_*i*_, β_*i*_ are drawn from some specified probability distribution ρ_α, β_(α, β). As a function of *x* the curve *f*(α_*i*_*x* + β_*i*_) is typically referred to as the tuning curve of neuron *i*. The output of these neurons is the sum of their weighted firing rates:

(1) $${\hat{g}}_{N}(x)=\sum_{i=1}^{N}\phi_{i}f(\alpha_{i}x+\beta_{i}).$$

Thus, the network takes any input *x* belonging to the appropriate space, and transforms it into some function ĝ_*N*_(*x*). If for example we wanted to compute the function *g*(*x*), we would need to pick ϕ_*i*_ such that ĝ_*N*_(*x*) ≈ *g*(*x*). The ϕ_*i*_ are referred to as the linear decoders in the NEF approach (Eliasmith and Anderson, 2004). They can be determined by minimizing the the following functional with respect to ϕ over some region *X* in *x* (Salinas and Abbott, 1995; Eliasmith and Anderson, 2004):

(2) $$\begin{array}{l} C(\phi )=\int_{X}({\hat{g}}_{N}(x)-g(x){)}^{2}dx+\lambda \sum_{i=1}^{N}\phi_{i}^{2}\\ \;=\int_{X}{\left(\sum_{i=1}^{N}\phi_{i}f(\alpha_{i}x+\beta_{i})-g(x)\right)}^{2}dx+\lambda \sum_{i=1}^{N}\phi_{i}^{2} \end{array}$$

where the first term in 2 corresponds to the error in the approximation and the second term penalizes large ϕ_*i*_. Minimizing *C*(ϕ) for ϕ yields the following linear system of equations:

(3) $$\Phi =A^{-1}\Gamma$$

(4) $$A_{ij}=\int_{X}f(\alpha_{i}x+\beta_{i})f(\alpha_{j}x+\beta_{j})dx+\delta_{ij}\lambda$$

(5) $$\Gamma_{j}=\int_{X}f(\alpha_{j}x+\beta_{j})g(x)dx.$$

Equations (3–5) correspond to standard function approximation (Bishop, 1995), although the basis functions *f* are randomly drawn. We will refer to the optimal decoders as Φ and any other decoder as ϕ. There are various functions *f* that have appeared in the literature. These are derived from complicated neural models using topological normal form theory (Ermentrout and Kopell, 1986; Izhikevich, 2007), are fits to experimental data from real neurons (Shriki et al., 2006), or are analytically derived from integrate and fire neurons. The general form the integrate-and-fire models we will consider is given by

(6) $$\dot{v}=F(v)+I$$

(7) $$v(t^{-})=v_{peak},\;\to v(t^{+})=v_{reset}$$

(8) $$f(I)=\{\begin{array}{ll} {\left(\int_{v_{reset}}^{v_{peak}}\frac{dv}{F(v)+I}\right)}^{-1} & I>0\\ 0 & I<0 \end{array}.$$

Specific examples include:

(9) $$F(v)=-\frac{v}{\tau_{v}}$$

(Leaky Integrate-and-Fire Model (Lapicque, 1907 Abbott, 1999; Brunel and Van Rossum, 2007)

(10) $$F(v)=v^{2}\text{ (Quadratic Integrate-and-Fire Model)}$$

(Izhikevich, 2003, 2007)

(11) $$F(v)=v^{2},\;v_{reset}=-\infty ,\;v_{peak}=\infty \text{ (Theta Model)}$$

(Ermentrout and Kopell, 1986)

(12) F(v)=exp(v)−v (Exponential-Integrate-and-Fire Model)

(Brette and Gerstner, 2005; Naud et al., 2008).

Other FI curves are fits to the measured FI curves of more sophisticated conductance based models or experimental measurements. For example, the function

(13) $$f(I)=\{\begin{array}{ll} I+c & I>0\\ 0 & I<0 \end{array}$$

can be fit to type-II firing rates when *c* > 0, and can be shown to be the steady state firing rate for neurons that display spike frequency adaptation when *c* = 0 (Ermentrout, 2006). Equation (13) has also been fit to conductance based models (Shriki et al., 2006) (with *x* = 0) and adequately describes the FI curves for many real cortical neurons (Stafstrom et al., 1984; Azouz et al., 1997; Ahmed et al., 1998).

As *x* is often thought of as a real world input variable in the NEF approach, the α_*i*_, β_*i*_ distribution can only be known once one specifies a distribution of maximal firing rates, *r*_*i*_ and *x*-intercepts, *a*_*i*_ for the tuning curves. For the time being, we will restrict the variable *x* to the interval [−1, 1]. It can be rescaled to an arbitrary interval, so this is no loss of generality. We will show later how *x* can also be extended to a vector as in the original NEF framework (Eliasmith and Anderson, 2004; Eliasmith, 2005). Once one specifies the distribution of (*r*_*i*_, *a*_*i*_), one can obtain a transformation of random variables. First, let us consider a population of neurons such that the maximal firing rate is achieved at *x* = 1:

$$\begin{array}{l} r_{i}=f(\alpha_{i}+\beta_{i})\\ \;0=\alpha_{i}a_{i}+\beta_{i} \end{array}$$

We will refer to these neurons as ON neurons as the neurons can either increase in firing rate with respect to *x* (ON neurons) or decrease (OFF neurons). The maximal firing rate for the ON population is reached at *x* = 1 (Eliasmith and Anderson, 2004) while the maximal firing rate for the OFF population is reached at *x* = −1. To generate a population of OFF neurons, we can reflect the tuning curves in the *x* = 0 axis by multiplying α_*i*_ by −1, which yields the following pair of transformations:

(14) $$\alpha_{i}=\pm \frac{f^{-1}(r_{i})}{1-a_{i}}$$

(15) $$\beta_{i}=-\frac{a_{i}f^{-1}(r_{i})}{1-a_{i}}$$

where the ± indicates ON/OFF, respectively and by *f*^−1^(*x*). Note that one can generate a population of ON and OFF neurons in different ways, for example by reflecting about the *x* = *a*_*i*_ axis for each neuron. We will treat *a*_*i*_ and *r*_*i*_ as our primary sources of heterogeneity in the case of approximating a function of a single variable and we will assume that the marginal densities are given by ρ_*a*_(*a*) and ρ_*r*_(*r*). Furthermore, we will write $a_{i}^{\pm }$ and $r_{i}^{\pm }$ to distinguish between the heterogeneous parameters for the ON(+) and OFF(−) populations. In this case, we can rewrite the sum (1) as

(16) $$\begin{array}{l} {\hat{g}}_{N}(x)=\sum_{i=1}^{N/2}\phi_{i}^{+}f\left(f^{-1}(r_{i}^{+})\left(\frac{x-a_{i}^{+}}{1-a_{i}^{+}}\right)\right)\\ \;+\sum_{i=1}^{N/2}\phi_{i}^{-}f\left(f^{-1}(r_{i}^{-})\left(\frac{-x-a_{i}^{-}}{1-a_{i}^{-}}\right)\right) \end{array}$$

where the first half represents the population of ON neurons and the second half of the sum represents the population of OFF neurons. Note that when we refer to the heterogeneous parameters $r_{i}^{max}$ and *a*_*i*_ for the ON and OFF populations, we do not imply that they have the same value for both populations for *i* = 1, 2, …*N* and they should have a superscript ± to denote which population the parameter belongs to. We do not include this superscript and note the abuse of notation for readers.

**Figure 1:**
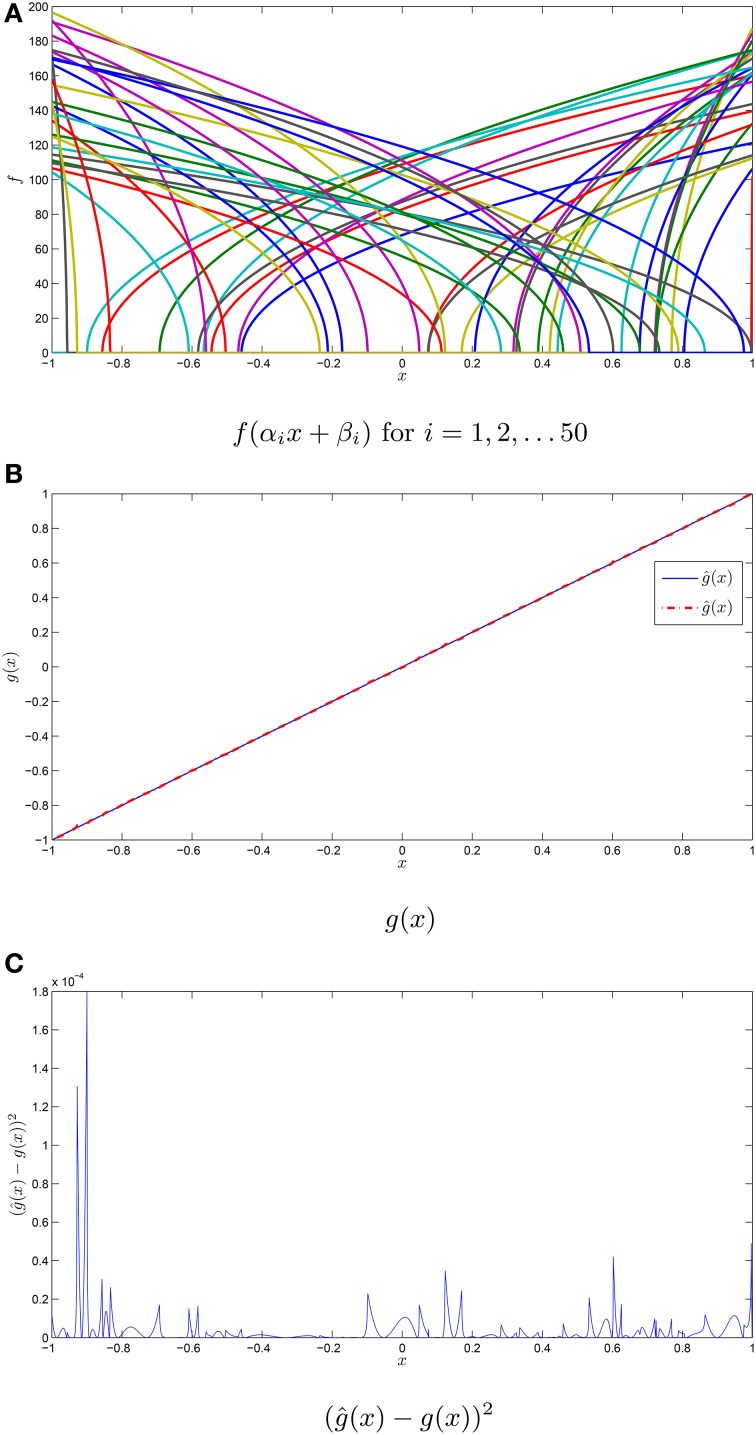
**Function approximation with neuronal tuning curves. (A)** The tuning curves for a population of 50 quadratic integrate and fire neurons with their intercepts and maximum firing rates drawn from independent uniform random variables on [−1, 1] and [100, 200]. The maximum firing rate is in Hz. **(B)** The function *g*(*x*) = *x*, in addition to the approximation ĝ(*x*) using the tuning curves from **(A)**. **(C)** The squared error in the approximation ĝ(*x*).

Suppose, for example, we wanted to approximate the function *g*(*x*) = *x* using a population of 50 quadratic integrate and fire tuning curves with 25 ON and 25 OFF neurons. This is shown in Figure 1 where the decoders are given by Equation (5). Note that reasonable accuracy is achieved despite the small population of neurons.

So far this has been fairly standard function approximation with a non-orthogonal basis (Bishop, 1995). The difference in the NEF approach is that one uses these linear decoders obtained from the firing rate curves to design a network of spiking neurons and the function is represented in the output of the network simulation. For example, the differential equation for the quadratic integrate and fire model is given by

(17) $${\dot{v}}_{i}=v_{i}^{2}+\alpha_{i}x+\beta_{i}$$

where if *v*(*t*^−^) = ∞, *v*(*t*^+^) = −∞. This can be written as the equivalent θ model with the transformation *v* = tan(θ/2) yielding:

(18) $${\dot{\theta }}_{i}=1-\cos(\theta_{i})+(1+\cos(\theta_{i}))(\alpha_{i}x+\beta_{i})$$

which produces a spike when θ(*t*^−^) = π and is reset to θ(*t*^+^) = −π.

Each of these differential equations generates a sequence of action potentials at specific spike times, *t*_*jk*_ where *t*_*jk*_ is the time of the *k*th spike fired by the *j*th neuron. These spike times are then fed into a post-synaptic filter *s*(*t*);

(19) $$\dot{s}(t)=-\frac{s(t)}{\tau_{s}}+\frac{1}{\tau_{s}}\sum_{j=1}^{N}\sum_{t_{jk}<t}\phi_{j}\delta (t-t_{jk}).$$

The linear decoders, ϕ_*j*_ are used to weight the spikes for their respective neuron. This post-synaptic filter equation can be explicitly integrated to yield:

(20) $$s(t)=\sum_{j=1}^{N}\sum_{t_{jk}<t}\phi_{j}\exp\left(\frac{t_{jk}-t}{\tau_{s}}\right)=\sum_{j=1}^{N}\sum_{t_{jk}<t}\phi_{j}E(t-t_{jk})$$

where *E*(*t*) = exp(−*t*/τ_*s*_). The integrated spike train for the *j*th neuron is approximately equal to its tuning curve, *f*(α_*j*_*x* + β_*j*_):

$$\int_{0}^{t}\sum_{t_{jk}<t}\delta (t-t_{jk})dt\approx \int_{0}^{t}f(\alpha_{j}x+\beta_{j})dt,$$

provided that *x* varies on a suitably slow time scale (Dayan and Abbott, 2001; Eliasmith and Anderson, 2004). In this case, the dynamics in Equation (19) are approximately given by

(21) $$s^{\prime }=-\frac{s}{\tau_{s}}+\frac{1}{\tau_{s}}\sum_{j=1}^{N}\phi_{j}f(\alpha_{j}x+\beta_{j})$$

This allows one to approximate an arbitrary dynamical system (Eliasmith, 2005). For example, if we consider a recurrent network (*x* = *s*), then to approximate the dynamics *s*′ = *F*(*s*) we merely require

(22) $$\sum_{i=1}^{N}\phi_{i}f(\alpha_{i}s+\beta_{i})\approx s+\tau_{s}F(s)={\hat{g}}_{N}(s)$$

and where the ϕ_*i*_ are given by Equation (3). Returning to the neural equations, if we take *x* = *s* and consider a recurrently coupled network of neurons then we have the following:

(23) $${\dot{v}}_{i}=F(v_{i})+\alpha_{i}s+\beta_{i}$$

(24) $$=F(v_{i})+\alpha_{i}\sum_{j=1}^{N}\sum_{t_{jk}<t}\phi_{j}E(t-t_{jk})+\beta_{i}$$

(25) $$=F(v_{i})+\sum_{j=1}^{N}\sum_{t_{jk}<t}\omega_{ij}E(t-t_{jk})+\beta_{i}$$

where ω_*ij*_ = α_*i*_ϕ_*j*_ is the NEF equation for the weight coupling neuron *j* to neuron *i* (Eliasmith and Anderson, 2004; Eliasmith, 2005) and the quantity

$$I_{syn,i}=\sum_{i=1}^{N}\sum_{t_{jk}<t}\omega_{ij}E(t-t_{jk})+\beta_{i}$$

is the post-synaptic current going to the *i*th neuron.

For example, if we wanted the macroscopic dynamics to be exponential decay, *F*(*x*) = *ks*, then we require ĝ(*s*) = *s*(1 + τ_*s*_*k*). We would obtain the ϕ_*i*_ by using Equations (3–5) which yields the optimal decoders Φ_*i*_ for ĝ(*x*) = *x*(1 + τ_*s*_*k*) and simulate our spiking network using the weights ω_*ij*_ = α_*i*_Φ_*j*_. This yields a recurrently coupled spiking neural network with macroscopic dynamics *s*′ = *ks*.

**Figure 2:**
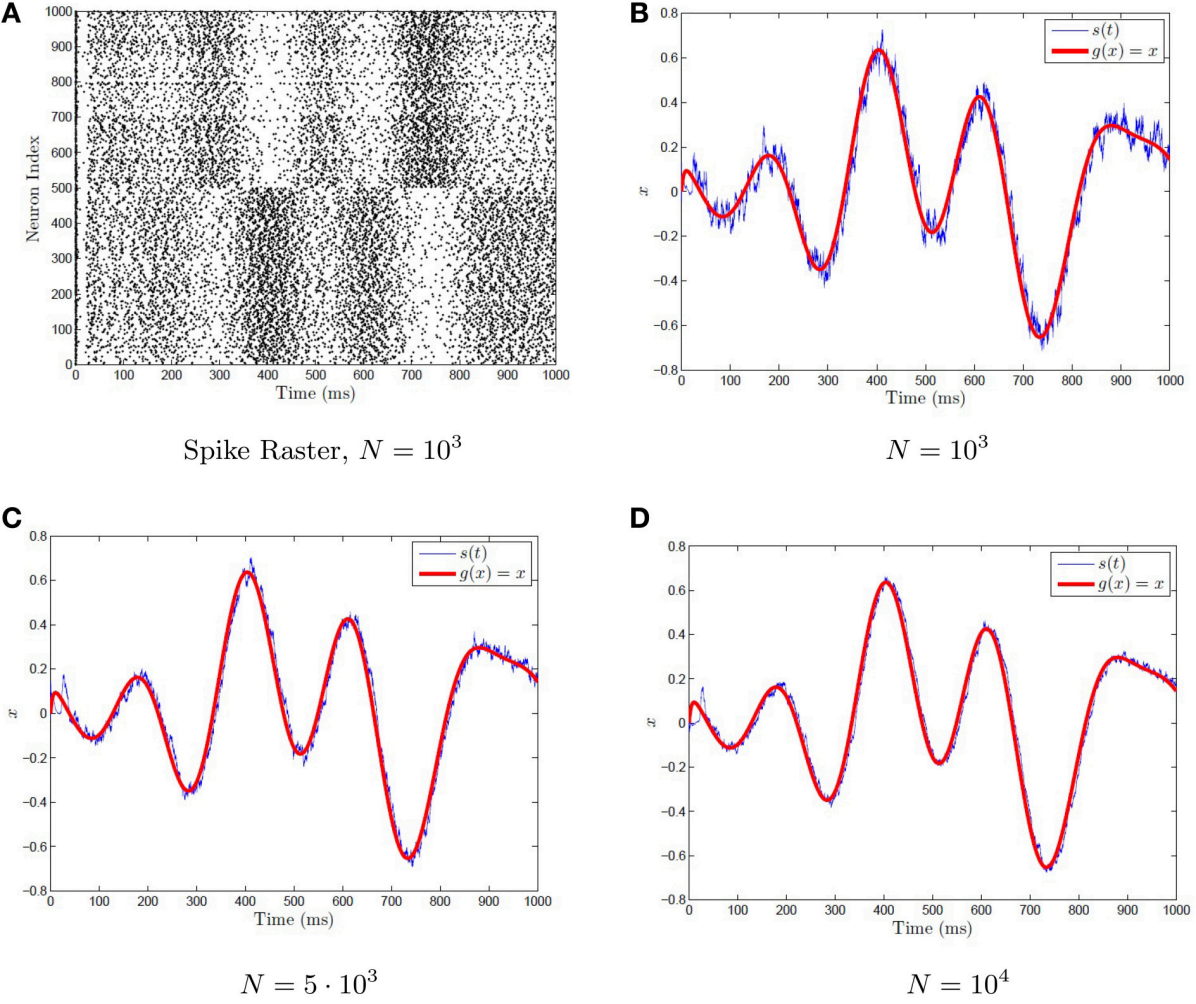
**Representation with a spiking neuronal network. (A)** A sample raster plot of a neural network with 1000 neurons performing representation. **(B–D)** The representation problem is approximated by networks of various sizes by imposing the condition (50). The neurons are quadratic integrate-and-fire neurons given by Equation (32). The spike train for **(B)** is plotted in **(A)**. A time varying randomly generated signal (red) is fed into the network, and is computed via the synaptic current variable *s*(*t*) using Equation 36 (blue). As the network size increases, the approximation becomes better.

In addition to recurrent networks, one can also construct feedforward networks with the NEF approach. For example, we can also treat *x* as an input variable. This allows a network to represent an input variable *x* in terms of its spiking. If τ_*s*_ is not large, then one can represent the input variable *x* as a postsynaptic current *s*:

(26) $$s\approx \sum_{i=1}^{N}\phi_{i}f(\alpha_{i}x+\beta_{i})={\hat{g}}_{N}(x)\approx x$$

assuming that *x* varies on a suitably slow time scale (slower than τ_*s*_). This is shown for example with networks of various sizes in Figure 2, with a synaptic time constant of τ_*s*_ = 5 ms approximating the function *g*(*x*) = *x*. The network of differential equations for the neurons is simulated using Equation (17). These neurons then generate a spike train which is weighted by the decoders. The weighted spike train is fed into the post-synaptic current variable *s*(*t*), which acts as the approximation for *g*(*x*) = *x*. A time varying *x*(*t*) is used that varies on a suitably slow time scale.

It is clear that given the fact that arbitrary functions or dynamics (via recurrent networks) can be computed, then one can generate multiple networks that perform different functions, and feed into one another. In this way, one could create large networks composed of interconnected subnetworks that perform functions such as controlling limbs, detecting objects, and performing tasks by using the mathematical approaches that already exist for accomplishing these feats and translating them into an equivalent neural network representation. This is the core idea in the NEF (Eliasmith and Anderson, 2004; Eliasmith et al., 2012).

Although a network of *N* = 100 neural tuning curves *f*(α_*i*_*x* + β_*i*_) is sufficient for a good approximation of many functions, depending on the dynamics being computed, significantly more neurons are needed in spiking simulations, as shown in Figure 2. Hundreds, if not thousands, of neurons are necessary for adequate approximation when spikes are used. The network size becomes even larger when we want to perform complicated functions involving more then one variable *x* or functions with higher frequency oscillations present. As the decoders are determined by large matrix inversion (Equation 3), this can take quite a while when dealing with more then 5000 neurons on a conventional computer. Furthermore, the smaller the synaptic time constant τ_*s*_, the more neurons are required. This is due to the fact that Equation (21) is effectively a kernel density estimator of the firing rate and when the bandwidth is too small, the resulting estimate is under-smoothed, thus requiring more neurons for a comparable degree of accuracy as that of a network with larger τ_*s*_. Furthermore, as the NEF weights are numerically determined (via the NEF decoders), the possible analysis is very limited. Thus, an analytical solution to the NEF decoders (and thus weights) would allow a greater insight into large networks and may also facilitate faster numerics.

### 2.2. Decoder asymptotics as *N* → ∞

**Figure 3:**
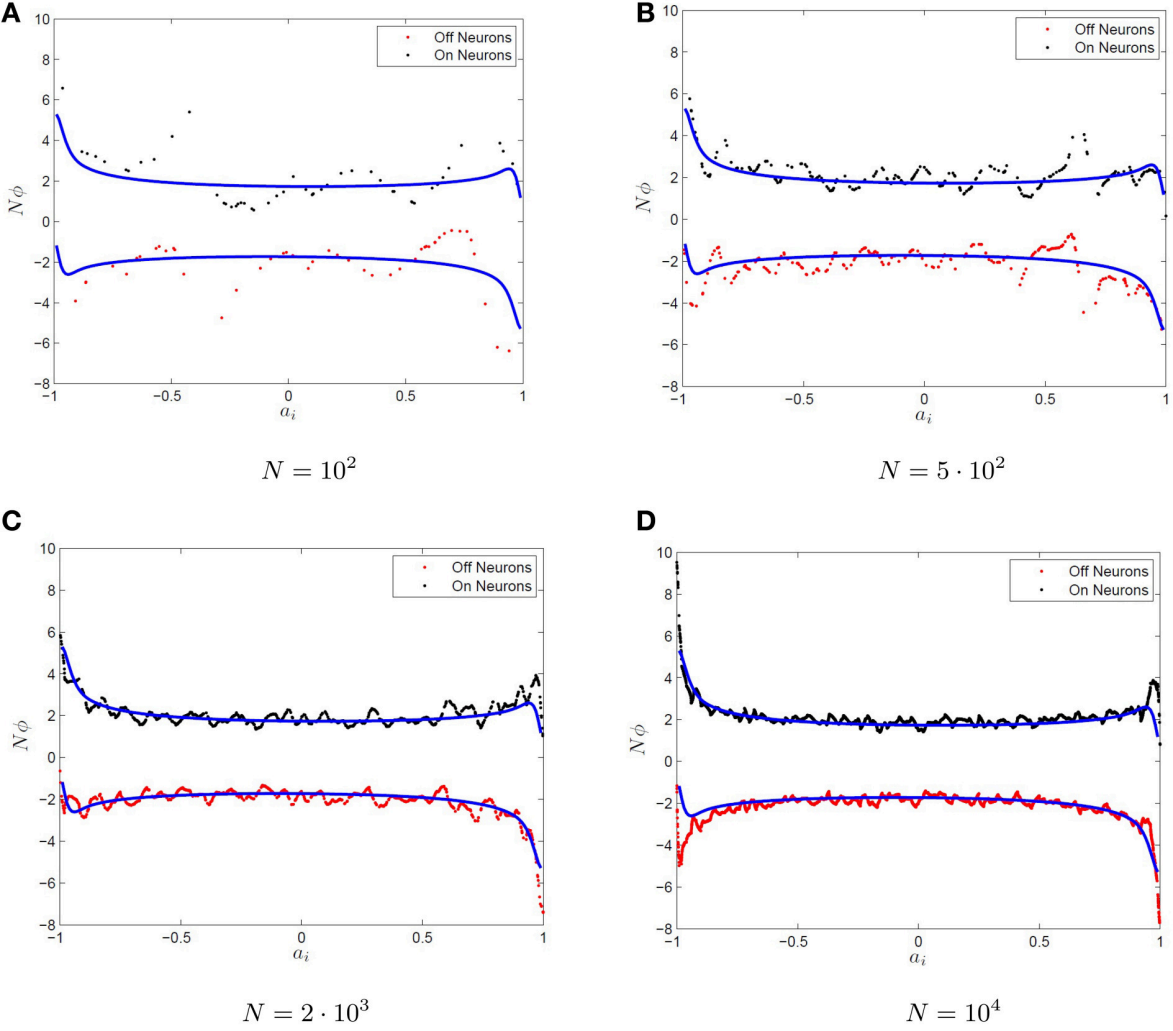
**Convergence of the optimal decoders to an invariant surface. (A–D)** The function *g*(*x*) = *x* is approximated by networks of various sizes using Equation (17) with firing rate curves. Plotted are the optimal decoders Φ_*i*_ scaled up by the network size *N* for ON (black dots) and OFF (red dots) neurons as a function of the intercept, *f*(0) for QIF firing rate functions. The quantity *NΦ*_*i*_ appears to converge as *N* → ∞ to the blue curve. The blue curve is determined by using a uniform grid of neurons over the heterogeneous parameter *a*_*i*_ and optimizing for the resulting decoders.

In order to proceed analytically, we will first look at the behavior of the optimal decoders Φ for large networks (*N* → ∞). To facilitate plotting, let us consider the case where for an arbitrary neural model, $f^{-1}\left(r_{i}^{\pm }\right)=\pm 1-a_{i}^{\pm }$, that is the maximim firing rate is given by $r_{i}^{\pm }=f\left(\pm 1-a_{i}^{\pm }\right)$, which reduces the random variables associated with the heterogeneity to the intercept variable, $a_{i}^{\pm }$. Additionally, note that for Type-I neurons:

(27) $$\begin{array}{l} f\left(\frac{f^{-1}(r_{i}^{\pm })}{1-a_{i}^{\pm }}(\pm x-a_{i}^{\pm })\right)=\sqrt{\frac{r_{i}^{\pm }{}^{2}}{1-a_{i}^{\pm }}(\pm x-a_{i}^{\pm })}\\ \;=\frac{r_{i}^{\pm }}{\sqrt{1-a_{i}^{\pm }}}\sqrt{\pm x-a_{i}^{\pm }} \end{array}$$

which immediately implies that we can absorb the quantitiy $\frac{r_{i}^{\pm }}{\sqrt{1-a_{i}^{\pm }}}$ into the decoder $\phi_{i}^{\pm }$ and rescale any solution we obtain by this quantity at the end. With Equation (27), the sum in Equation (16) becomes:

(28) $${\hat{g}}_{N}(x)=\sum_{i=1}^{N/2}\Phi_{i}^{+}f(x-a_{i}^{+})+\sum_{i=1}^{N/2}\Phi_{i}^{-}f(-x-a_{i}^{-})$$

where the Φ_*i*_ are determined by Equation (5). One should note that there is an abuse of notation here, as the optimal decoders differ for the ON/OFF subpopulations for *i* = 1, 2, …*N* however we have used the same symbol to denote the optimal decoders, Φ_*i*_ for both populations. Additionally, the value $a_{i}^{+}$ is the threshold to firing for the *i*th ON neuron while the quantity $-a_{i}^{-}$ is the threshold to firing for the *i*th off neuron. If one were to plot the decoders for large *N*, then one can easily see that in the limit of large network size (*N* → ∞), the individual decoders vanish (Φ_*i*_ → 0, not shown here). However, for increasing *N*, it seems that the quantity γ_*i*_ = *NΦ*_*i*_/2 converges to some non-zero value γ(*a*_*i*_) and thus it appears that γ_*i*_ converges to some function of the *x*-intercept, *a*_*i*_, the source of heterogeneity for the neurons. This is shown in Figure 3 for increasingly large networks. The quantity *NΦ*_*i*_/2 is plotted vs. *a*_*i*_. The predicted surface for convergence, γ(*a*), is also plotted which is determined by optimizing over a uniform mesh in the parameter space. We will refer to any γ_*i*_ that satisfies γ_*i*_ = ϕ_*i*_*N*/2 for some decoder ϕ_*i*_ as *scale invariant decoders* and γ^±^(*a*) as the *decoder surface*. We will not necessarily use the same decoders for the ON and OFF neurons hence the superscipt on γ(*a*). We will show in the subsequent sections how to determine the decoder surfaces for the type-I and type-II (approximate) firing rates for single variable and multivariable functions.

In order to determine the decoder surface analytically, we need to understand the behavior of the network as *N* → ∞. Using the scale invariant decoders from Equation (28):

(29) $$\begin{array}{l} {\hat{g}}_{N}(x)=\sum_{i=1}^{N/2}\phi_{i}^{+}f(x-a_{i}^{+})+\sum_{i=1}^{N/2}\phi_{i}^{-}f(-x-a_{i}^{-})\\ \;=\frac{2}{N}\sum_{i=1}^{N/2}\gamma^{+}(a_{i}^{+})f(x-a_{i}^{+}) \end{array}$$

(30) $$\;+\frac{2}{N}\sum_{i=1}^{N/2}\gamma^{-}(a_{i}^{-})f(-x-a_{i}^{-})$$

(31) $$\;=\bar{\gamma_{i}^{+}f(x-a_{i}^{+})}+\bar{\gamma_{i}^{-}f(-x-a_{i}^{-})}$$

where the overline denotes the finite average over the inhomogeneity in the intercepts. We should expect that as *N* → ∞, the finite network averaging turns into an expectation:

(32) $$\begin{array}{l} \bar{\gamma_{i}f(x-a_{i}^{+})}\to E(\gamma^{+}(a_{i}^{+})f(x-a_{i}^{+}))\\ \;=\int_{-1}^{x}\gamma^{+}(a)\rho_{a}(a)f(x-a)da \end{array}$$

(33) $$\begin{array}{l} \bar{\gamma_{i}f(-x-a_{i}^{-})}\to E(\gamma^{-}(a_{i}^{-})f(-x-a_{i}^{-}))\\ \;=\int_{-1}^{-x}\gamma^{-}(a)\rho_{a}(a)f(-x-a)da \end{array}$$

where ρ_*a*_(*a*) is the probability density describing the heterogeneity variable *a*_*i*_ for the neurons. For the Equations (32, 33) to be valid for the optimal decodoers, we would need to formally show that Φ_*i*_ → 2γ(*a*_*i*_)/*N*, where Φ_*i*_ are the optimal decoders for some particular scale-invariant decoder γ(*a*_*i*_). However, this is unnecessary as we can regard ϕ_*i*_ = 2γ(*a*_*i*_)/*N* as a suboptimal decoder and independent of the optimal decoders which are generated by minimizing the integral Equation (2). In which case, the limit exists by the law of large numbers. Furthermore, as we shall show later, Φ_*i*_ will not necessarily converge to γ(*a*_*i*_) as γ(*a*_*i*_) is non-unique, where as Φ_*i*_ is the optimal decoder which is unique due to the quadratic error surface in *C*(ϕ).

Note that γ(*a*)ρ_*a*_(*a*) appears as a product in the integral. These terms can be collapsed into a single function ${\hat{P}}^{\pm }\left(a\right)=\gamma^{\pm }\left(a\right)\rho_{a}\left(a\right)$. We will refer to these quantities as the *weighted decoders* of the ON/OFF neurons and use the weighted decoders to define the linear operators;

(34) $$L^{+}({\hat{P}}^{+})=\int_{-1}^{x}{\hat{P}}^{+}(a)f(x-a)da={\hat{g}}^{+}(x)$$

(35) $$L^{-}({\hat{P}}^{-})=\int_{-1}^{-x}{\hat{P}}^{-}(a)f(-x-a)da={\hat{g}}^{-}(x)$$

(36) $$M({\hat{P}}^{+},{\hat{P}}^{-})=L^{+}({\hat{P}}^{+})+L^{-}({\hat{P}}^{-})=\hat{g}(x).$$

which we will refer to as the *tuning curve transforms* (TCT). The TCTs map functions from the space of the variable(s) assigned to the heterogeneous parameters to the space of functions we are trying to approximate. Note that these operators are actually applied to different weighted decoders as the decoder surfaces are different for ON and OFF neurons. Furthermore, the density ρ_*a*_(*a*) need not be identical for both ON and OFF neurons. However, in all numerical implementations, ρ_*a*_(*a*) will be identical for the sake of simplicity.

Suppose we could determine $\hat{P}\left(a\right)$ analytically. In this case, as $\gamma \left(a\right)\rho_{a}\left(a\right)=\hat{P}\left(a\right)$, whenever ρ_*a*_(*a*) ≠ 0, we can compute $\gamma \left(a\right)=\hat{P}\left(a\right)/\rho_{a}\left(a\right)$ and leave γ(*a*) undefined otherwise (as there is no neuron that has parameter(s) in this region). Now, given the fact that we obtain a linear operator as *N* → ∞ case, the real problem becomes in finding the $\left({\hat{P}}^{+},{\hat{P}}^{-}\right)$ such that $M\left({\hat{P}}^{+},{\hat{P}}^{-}\right)$ maps to *g*(*x*), the function we want to approximate. That is, we have to invert the operator *M* for these $\hat{P}$. If we know these $\hat{P}$, then as we presumably know the distribution of tuning curve intercepts, we can determine the decoders ϕ_*i*_ with:

(37) $$\phi^{\pm }(a_{i}^{\pm })=\frac{2\gamma^{\pm }(a_{i}^{\pm })}{N}=\frac{2}{N}\frac{{\hat{P}}^{\pm }(a_{i}^{\pm })}{\rho_{a}(a_{i}^{\pm })}$$

and thus the analytically determined scale-invariant decoders γ(*a*_*i*_) are effectively weights for a Monte Carlo estimate of the integral operator TCTs.

Here, we will explicitly invert the tuning curve transforms for single variable functions in Section 2.3. The resulting equation for the weighted decoders is a convolution integral. In Section 2.4 we will show that with a basis to basis mapping, one can also invert the tuning curve transforms for multi-variable functions.

### 2.3. Single variable functions

If we work with the operators *L*^+^ and *L*^−^ separately, the problem becomes entirely tractable. One of the surprising things about the operators *L*^+^ and *L*^−^ is that provided that the functions we are considering are constrained to a subset where *g*^+^(*x*) vanishes to first order at *x* = −1 and *g*^−^(*x*) vanishes to first order at *x* = 1, and are both smooth, then the operators are invertible analytically on this constrained subspace of functions using Laplace transforms (see Appendix). Additionally, by using both ${\hat{P}}^{+}\left(a\right)$ and ${\hat{P}}^{-}\left(a\right)$, one can compute any smooth function irrespective of the conditions at *x* = ±1. Furthermore, piecewise smooth continuous functions can also be computed (see Supplementary Materials). Closed form solutions do not exist for all neuronal firing rates as the Laplace transform cannot always be inverted explicitly. However, the type-I and type-II firing rate models do have analytically determined decoders. For the type-I/theta neuron firing rate, we have (see Appendix for derivation):

(38) $$\begin{array}{l} {\hat{P}}^{+}(a)=\frac{2}{\pi }\left(\frac{g(-1)+g(1)}{2}+g^{\prime }(-1)\right)\frac{1}{\sqrt{1+a}}\\ \;+\int_{0}^{a+1}g^{\prime \prime }(a-t)\frac{2}{\pi \sqrt{t}}dt \end{array}$$

(39) $$\begin{array}{l} {\hat{P}}^{-}(a)=\frac{2}{\pi }\left(\frac{g(-1)+g(1)}{2}-g^{\prime }(1)\right)\frac{1}{\sqrt{1+a}}\\ \;+\int_{0}^{a+1}g^{\prime \prime }(t-a)\frac{2}{\pi \sqrt{t}}dt \end{array}$$

**Figure 4:**
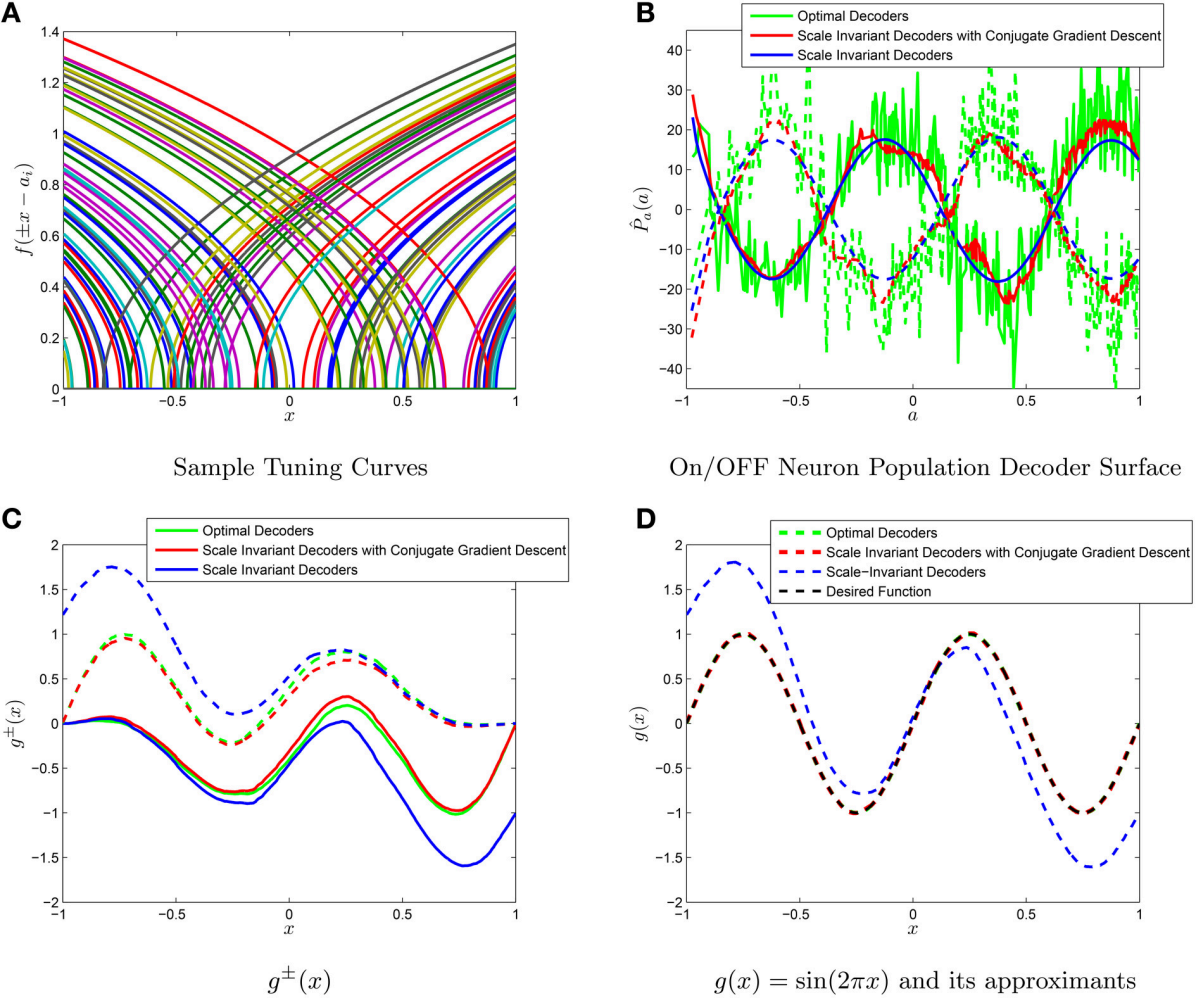
**Comparison of the scale-invariant decoders and the NEF decoders. (A)** A sample subset of tuning curves from a population of 1000 neurons. The function *g*(*x*) = sin(2π*x*) is approximated using populations of *N* = 10^3^ ON and OFF neurons. **(B)** The scale invariant decoders (blue), the scale invariant decoders with conjugate gradient descent fine-tuning (red) and the optimal decoders multiplied by *N* (green) for both the ON (solid) and OFF (dashed) groups of neurons. **(C)** The different decoders correspond to different *g*^±^(*x*). **(D)** Summing the *g*^±^(*x*) yields approximations to *g*(*x*). The conjugate gradient descent improves the approximation (from 6^*^10^−1^ to 7^*^10^−5^) of magnitude while still maintaining a tight correlation with the scale-invariant decoders (*p* = 0.9811). The optimal decoders have a mean-squared error of 9^*^10^−7^ for comparison purposes.

The weighted decoder solutions for the type-II firing rate are also contained in the Appendix. For example, we have approximated the function *g*(*x*) = sin(2π*x*) using 10,000 type I tuning curves, as shown in Figure 4. An important thing to notice is the linearity in Equations (38,39) and (36) in the target function *g* and thus linearity for the scale-invariant decoders γ(*a*). Furthermore, due to the fact we have considered ${\hat{P}}^{\pm }\left(a\right)$ to be separate for the ON and OFF populations, our operator for determining *g*(*x*) is $ĝ\left(x\right)=M\left({\hat{P}}^{+},{\hat{P}}^{-}\right)=L^{+}\left({\hat{P}}^{+}\right)+L^{-}\left({\hat{P}}^{-}\right)$. However, while our range in *L*^+^ and *L*^−^ was constrained, it was not constrained enough to provide a unique solution to *M*(*P*^+^, *P*^−^) = *g*(*x*). In particular, if we consider any function ϵ(*x*) that lies in both admissable spaces (vanishes to first orders at *x* = 1 and *x* = −1), then ϵ(*x*) can be represented by both populations with ${\hat{P}}_{\epsilon }^{\pm }\left(a\right)$, respectively and thus

(40) $${\tilde{P}}_{g(x)+\epsilon (x)}^{+}(a)={\hat{P}}_{g(x)}^{+}(a)+{\hat{P}}_{\epsilon (x)}^{+}(a)$$

(41) $${\tilde{P}}_{g(x)-\epsilon (x)}^{-}(a)={\hat{P}}_{g(x)}^{-}(a)-{\hat{P}}_{\epsilon (x)}^{+}(a)$$

are also valid solutions to *M*(*P*^+^, *P*^−^) = *g*(*x*). One can interpret this as a degree of freedom in terms of the decoders (and thus the synaptic weights). For example, we can use *e*(*x*) to minimize the expected squared error or other criterion. Thus, in the following we will strictly assume that *e*(*x*) = 0.

We should note that it is possible to numerically invert the Laplace transforms resulting from the derivation process for the other firing rate curves. However, for now we will primarily work with the type I and type II curves. Our numerics will also primarily consist of networks of theta neurons (type-I).

#### 2.3.1. Convergence rate for single-variable functions

With our decoder surfaces in hand, we can now proceed to determine the various convergence properties in the limit as *N* → ∞. In particular, we have the following:

(42) $$\begin{array}{l} E_{a}\left((g(x)-{\hat{g}}_{N}(x))2\right)=\frac{2}{N}(\begin{array}{l} \begin{array}{l} \int \begin{array}{c} x\\ -1 \end{array}\;\gamma^{+}{\left(a\right)}^{2}\rho_{a}(a){\left(x-a\right)}^{2}da\\ \;-\;g^{+}{\left(x\right)}^{2} \end{array} \end{array})\\ \;+\frac{2}{N}(\begin{array}{l} \begin{array}{l} \int \begin{array}{c} x\\ -1 \end{array}\;\gamma^{-}{\left(a\right)}^{2}\rho_{a}(a)f{\left(-x-a\right)}^{2}da\\ \;-g^{-}{\left(x\right)}^{2} \end{array} \end{array}) \end{array}$$

which immediately implies that our approximant ĝ_*N*_(*x*) converges in mean-square to *g*(*x*) pointwise in *x* provided that:

(43) $$\int_{-1}^{1}\gamma^{\pm }{\left(a\right)}^{2}\rho_{a}(a)da=\int_{-1}^{1}\frac{{\hat{P}}^{\pm }{\left(a\right)}^{2}}{\rho_{a}(a)}da<\infty .$$

A derivation of Equation (42) can be found in the Supplementary Materials. Letting γ_*N*_ = (γ(*a*_*i*_)/*N*, …γ(*a*_*N*_)/*N*), then we can also consider how the distribution of the quadratic cost function *C*(γ_*N*_) scales as *N* → ∞ ; from Equation (2):

(44) $$\begin{array}{l} C(\gamma_{N})=\int_{X}{\left(\frac{1}{N}\sum_{i\;=\;1}^{N}\gamma^{e_{i}}(a_{i})f(e_{i}x-a_{i})-g(x)\right)}^{2}dx\\ \;+\;\frac{\lambda }{N^{2}}\sum_{i\;=\;1}^{N}\gamma {\left(a_{i}\right)}^{2} \end{array}$$

where *e*_*i*_ is the symbolic variable ± denoting the identify of a neuron as OFF/ON. One can show that provided that Equation (43) holds, and γ^±^(*a*) is bounded on [−1, 1], then we have the following

(45) $$E(C(\gamma_{N}))\text{​}\leq \text{​}O(N^{-1}),\text{​​ }E({\left(C(\gamma_{N})-E(C(\gamma_{N}))\right)}^{2})\leq O(N^{-2})$$

which implies that as *N* → ∞, then *C*(γ_*N*_) → 0 in a mean-square sense. This is proven in the Supplementary Materials. As the cost function is strictly positive, then we can interpret this as γ_*N*_ minimizing the cost asymptotically.

For other neuronal models, one can merely use the maximum firing rates and intercepts to approximate their tuning curves with the type I standard form or the linear firing rate tuning curves. This will yield scale-invariant decoders that can be used on the tuning curves for the actual neuronal model with some degree of error. Additionally, gradient descent algorithms can be used to refine the scale-invariant decoders and that take into account the systematic error in using the type-I/type-II tuning curve approximation.

### 2.4. Multivariable functions

It is clear that in the preceding section, one could approximate any arbitrary single variable function using scale invariant decoders. The same can be said about multi-variable functions. We will first introduce linear encoding for multi-variable inputs. In the NEF, it is assumed that the current input into each neuron takes the form:

(46) $$I_{i}=\alpha_{i}\langle e,x\rangle +\beta_{i}$$

where ***e*** is the encoding vector that lies on the n-dimensional unit sphere and ***x*** lies in the interior; 〈***e***, ***x***〉 is the standard dot-product. The maximum firing rate occurs when ***x*** = ***e***, and thus 〈***e***, ***x***〉 = 1 is the maximum. The vector ***e*** is also referred to as the preferred direction vector. In this case, there are no ON and OFF neurons as they are effectively taken care of by the angle in between ***x*** and ***e***. If ***x*** and ***e*** are colinear, then the maximum firing rate occurs when ***e*** = ***x***, and the firing rate is zero when 〈***e***, ***x***〉 = *a*, the equation for the hyperplane with normal vector ***e***. Note that because the unit sphere in one-dimension is merely ±1, we have a direct correspondence with the *e*_*i*_ from the single variable analysis in the previous section.

Once again, we can non-dimensionalize:

(47) $$f(\alpha_{i}\langle e_{i},x\rangle +\beta_{i})=f\left(\frac{f^{-1}(r_{i})}{1-a_{i}}(\langle e,x\rangle -a_{i})\right).$$

To simplify the situation, we will again assume that $f^{-1}\left(r_{i}\right)=1-a_{i}$, to remove this term. As before, for type-I firing rates this occurs without any loss of generality.

While it may seem like this setup complicated matters somewhat, in the limit that *N* → ∞, the end result is simpler then the single variable case as we can make use of the orthogonality of the trigonometric functions to derive an appropriate basis to basis mapping. Consider suboptimal decoders of the form $\phi =\frac{\gamma \left(e,a\right)}{N}=\frac{\gamma_{e}\left(e\right)\gamma_{a}\left(a\right)}{N}$. For a separable decoder we have:

$${\hat{g}}_{N}(x)=\frac{1}{N}\sum_{i=1}^{N}\gamma_{e}(e_{i})\gamma_{a}(a_{i})f(\langle e_{i},x\rangle -a_{i})$$

which in the large network limit becomes:

$$\begin{array}{l} {\hat{g}}_{N}(x)=\int_{\|e\|=1}\int_{-1}^{\langle e,x\rangle }\gamma_{e}(e)\gamma_{a}(a)f(\langle e,x\rangle -a)\rho_{e}(e)\\ \;\rho_{a}(a)da\;de \end{array}$$

$$=\int_{\|e\|=1}\int_{-1}^{\langle e,x\rangle }{\hat{P}}_{e}(e){\hat{P}}_{a}(a)f(\langle e,x\rangle -a)da\;de$$

From our previous work, we know that the weighted decoder ${\hat{P}}_{a}\left(a\right)$ can be chosen such that:

(48) $$\int_{-1}^{\langle e,x\rangle }{\hat{P}}_{a}(a)f(\langle e,x\rangle -a)da={\left(\langle e,x\rangle +1\right)}^{n}$$

by treating *z* = 〈***e***, ***x***〉 and using Equation (38) to determine $\hat{P}\left(a\right)$. The specific form of ${\hat{P}}_{a}\left(a\right)$ that performs this transformation varies from neural model to neural model. For the type I/type II firing rate, it is given by a recurrence relationship in terms of the binomial exponent *n* and is included in the Supplementary Materials. With ${\hat{P}}_{a}\left(a\right)$ determined, the decoders ${\hat{P}}_{e}\left(e\right)$ are characterized by the integral equation:

(49) $${\hat{g}}_{N}(x)=\int_{\|e\|=1}{\hat{P}}_{e}(e){\left(\langle e,x\rangle +1\right)}^{n}de$$

To proceed further, we will exploit the orthogonoality of the Fourier series in a hyper-spherical coordinate system. For example, in two-dimensions we have:

(50) $${\hat{g}}_{N}(x)=\int_{0}^{2\pi }{\hat{P}}_{\theta }(\theta ){\left(\cos(\theta )x+\sin(\theta )y+1\right)}^{n}d\theta$$

The second term in the integrand is a polynomial in cos(θ) and sin(θ). By DeMoivres formula, this can be expressed as a Fourier series with coefficients that depend on *x* and *y* where the series contains no cos(*mθ*) or sin(*mθ*) for *m* > *n*. Thus, we can extract out polynomial basis functions in the *x* and *y* using $\hat{P}\left(\theta \right)=\cos\left(m\theta \right)\sin\left(k\theta \right)$ for *m, k* < *n*. For example, the first few $\hat{P}\left(\theta \right)$ and the corresponding ĝ_*N*_(*x*) are shown in Table 1.

**Table 1:** **The basis-to-basis mapping for a polar coordinate system for the first few *n***.

**ĝ_*N*_(*x*)**	**$\hat{P}\left(\theta \right)$**	***n***
1	$\frac{1}{2\pi }$	1
*x*	$\frac{\cos\left(\theta \right)}{\pi }$	1
*y*	$\frac{\sin\left(\theta \right)}{\pi }$	1
*x*^2^+*y*^2^+2	$\frac{1}{\pi }$	2
$\frac{1}{2}\left(x^{2}-y^{2}\right)$	$\frac{sin\left(2\theta \right)}{\pi }$	2
*xy*	$\frac{\cos\left(2\theta \right)}{\pi }$	2

As we can obtain a polynomial basis where the maximum polynomial power is arbitrary, we can approximate any arbitrary integrable function. In general, one uses a sequence of trigonometric basis in the heterogeneous space to yield a polynomial basis in the function approximation space. One may wonder if the non-uniqueness in the scalar case was due to the peculiarities of the unit sphere in 1-dimension (an isolated pair of points). This turns out not be the case (see Supplementary Materials).

#### 2.4.1. Convergence rate for multivariable functions

One can again determine the order of convergence for the scale-invariant decoders for a multi-variable funciton. An application of the law of large numbers yields:

$$\begin{array}{l} E({\hat{g}}_{N}(x))=E\left(\gamma (e_{i})\gamma (a_{i})f(\langle e_{i},x\rangle -a_{i})\right)\\ \;=g(x) \end{array}$$

(51) $$\begin{array}{l} E(({\hat{g}}_{N}(x)-E\left({\hat{g}}_{N}(x)){)}^{2}\right)=E\left({\left({\hat{g}}_{N}(x)-g(x)\right)}^{2}\right)\\ \;=\frac{1}{N}\left[\begin{array}{l} E(\gamma {\left(e_{i}\right)}^{2}\gamma {\left(a_{i}\right)}^{2}f^{2}(\langle e_{i},x\rangle \\ \;-a_{i}))-g{\left(x\right)}^{2} \end{array}\right] \end{array}$$

and thus the expected square error converges like 1/*N* implying that ĝ_*N*_(*x*) converges to *g*(*x*) in mean-squared. The expectation is taken over the random variables ***e***_*i*_ and *a*_*i*_. As the convergence rate is somewhat slow, it is natural to ask whether or not it is possible to improve the the expected squared error.

While there are many analytical paths one may take, we leave these approaches for future work. We will primarily use gradient descent variants that do not require computing the Hessian. If we knew the Hessian, then for a finite network we could immediately solve the system of Equations (3–5) as the problem is entirely quadratic and can be resolved numerically with the Hessian matrix. However, solving the quadratic problem with the Hessian requires large matrix inversion, and this is simply not feasible for large networks. Thus, we can use Hessian-free gradient descent methods. For example, one can use various conjugate gradient type algorithms to improve the expected squared error significantly with only a few iterations, and no large matrix inversion. Additionally, one can use the methods of stochastic gradient descent, such as weight perturbation, and node perturbation (Werfel et al., 2005). We will primarily use conjugate-gradient descent implemented with the PCG function in MATLAB (MATLAB, 2014). The crucial thing about these approaches is that we can obtain substantial improvements to the expected squared error with only slight perturbations to the scale-invariant decoders, as we shall see when we look at specific examples.

## 3. Results

To simulate networks with arbitrary dynamics, we can use the decoders derived in the previous sections along with neurons that correspond to the appropriate firing rates (Eliasmith and Anderson, 2004). Suppose the variable **s**(*t*) represents a vector of decoded firing rates given by the following equation:

(52) $$s_{i}{\left(t\right)}^{\prime }=-\frac{s_{i}}{\tau_{s}}+\frac{1}{\tau_{s}}\sum_{j=1}^{N}\phi_{ji}\sum_{t_{j,k}<t}\delta (t-t_{j,k})$$

(53) $$s=-\frac{s}{\tau_{s}}+\frac{1}{\tau_{s}}\sum_{j=1}^{N}\phi_{j}\sum_{t_{j,k}<t}\delta (t-t_{j,k})$$

(54) $$\approx -\frac{s}{\tau_{s}}+\frac{1}{\tau_{s}}\sum_{j=1}^{N}\phi_{j}f(\langle e,s\rangle -a_{i})$$

Where **ϕ**_*j*_ is the decoder for the *j*th neuron. Equation (54) is referred to as the rate equation while Equation (52) is the equation for *s*_*i*_ under neuronal spiking with a simulated spiking neuronal network. The time constant used will be 50 ms unless otherwise stated.

Using the same procedure as before, by integrating the spiking equation for **s**(*t*) explicitly, one can derive the NEF equation for the synaptic weights:

(55) $$\omega_{ij}=\alpha_{i}\langle e_{i},\phi_{j}\rangle$$

where ω_*ij*_ is the synaptic weight for the post-synaptic neuron *i* and the presynaptic neuron *j* in a recurrent neuronal network and ϕ_*j*_ is the scale invariant decoder for the function

(56) $$F(s)\tau_{s}+s$$

For a scale invariant decoder, this yields the following synaptic weight:

(57) $$\omega_{ij}=\frac{1}{N\rho (a_{j})\rho_{e}(e_{j})}\frac{f^{-1}(r_{i})}{1-a_{i}}\langle e_{i},{\hat{P}}_{e}(e_{j}){\hat{P}}_{a}(a_{j})\rangle$$

Note that ω_*ij*_ = *F*(*a*_*i*_, *a*_*j*_, *r*_*i*_, *r*_*j*_, ***e***_*i*_, ***e***_*j*_), is a function of random variables for the presynaptic and post-synaptic neurons. Thus, instead of thinking the weights as a matrix of numerical values, or as a direct graph, one may think of the weights as defining a hypersurface in a higher dimensional space. For example, the formula (Equation 56) describes a hypersurface with 2*m* + 4 dimesions where *m* is the dimension of the dynamics the network simulates.

In the following examples, we will generate networks with these analytically determined weights using scale-invariant decoders that display the prescribed dynamics. Additionally, we will assume that

$$r_{i}^{max}=M\sqrt{1-a_{i}}$$

(58) $$\rho_{a}(a)=\frac{1}{2\sqrt{2}\sqrt{1+a}}$$

The variable *M* controls the maximum firing rate of the neurons, with the range of maximum firing rates being between $\left[0,\sqrt{2}M\right]$. We take *M* to be 60 Hz for all subsequent numerics, unless otherwise specified. Note that we need α_*i*_ to compute the weights. The α_*i*_ differs depending on whether or not we are simulating a scalar system or a multi-variable system. For a scalar system, $\alpha_{i}=M^{2}e_{i}$ where *e*_*i*_ = 1 for ON neurons and −1 for OFF neurons. For a vector, $\alpha_{i}=M^{2}$. For multi-variable dynamics we will assume uniform distributions in the hyperspherical coordinate systems. With the former assumption, the tuning curves for the neurons simply become $M\sqrt{\langle e,x\rangle -a}$ where the *M* can be absorbed into the decoder. We will generate networks of spiking theta neurons that simulate a neural integrator, a Van der Pol Oscillator, and the Lorenz system.

### 3.1. Example 1: neural integrator

A neural integrator is a recursively coupled neural network that integrates an incoming signal, *u*(*t*). The coupling variable *s*(*t*), will have dynamics given by

(59) $$s^{\prime }(t)=u(t)=-\frac{s}{\tau_{s}}+\frac{1}{\tau_{s}}\sum_{i=1}^{N}\phi_{i}\sqrt{e_{i}(s(t)+\tau_{s}u(t))-a_{i}}$$

where *e*_*i*_ = 1 if neuron *i* is an ON neuron and −1 for OFF neurons. Note that we have scaled *u*(*t*) by τ_*s*_ as this allows us to write:

(60) $$\tau_{s}u+s=\sum_{i=1}^{N}\phi_{i}\sqrt{e_{i}(s+\tau_{s}u)-a_{i}}.$$

We then require the scale invariant decoders such that $ĝ\left(z\right)=\overset{N}{\underset{i=1}{\sum }}\phi_{i}\sqrt{e_{i}z-a_{i}}\approx z$. A set of *g*^±^(*x*) and the corresponding scale-invariant decoders is given by:

(61) $$g^{+}(x)=\frac{1}{2}\left(1+x\right),\;g^{-}(x)=-\frac{1}{2}\left(1-x\right)$$

(62) $${\hat{P}}^{+}(a)=\frac{2}{\pi }\frac{1}{\sqrt{1+a}M},\;{\hat{P}}^{-}(a)=-\frac{2}{\pi }\frac{1}{\sqrt{1+a}M}$$

(63) $$\phi_{i}^{\pm }=\frac{\gamma^{\pm }(a)}{N}=e_{i}\frac{4\sqrt{2}}{NM\pi }$$

which yields ĝ(*z*) = *z*. From formula (14) for α_*i*_ and (58) for the density of *a*_*i*_, and we have $\alpha_{i}=e_{i}M^{2}$ and the neuronal weight

(64) $$\omega_{ij}=\alpha_{i}\phi_{j}=e_{i}e_{j}\frac{4\sqrt{2}M}{N\pi }.$$

All the synaptic weights here are given by $4\sqrt{2}M/\left(N\pi \right)$ for ON/ON and OFF/OFF connections and $-4\sqrt{2}M/\left(N\pi \right)$ for ON/OFF and OFF/ON connections. Now, we will exploit symmetry and non-uniqueness to generate two more neuronal integrators with the same initial distributions of heterogeneity ρ_*a*_(*a*). In particular, consider the function ϵ(*x*) = (1 − *x*^2^)^2^, this function lies in both function spaces for *g*^±^(*x*) as it vanishes to second order at both boundaries. Additionally, it can be computed using the following scale-invariant decoders:

(65) $${\hat{P}}_{\epsilon (x)}^{\pm }(a)=\frac{32\pi }{5M}(4a^{2}-2a-1)\sqrt{a+1}$$

(66) $$\phi_{i}\text{​}=\text{​}\frac{\gamma (a_{i}^{\pm })}{N}\text{​}=\text{​}e_{i}\frac{64\sqrt{2}}{5M\pi N}(1+a_{i}^{\pm })(4a^{\pm }{}_{i}^{2}-2a_{i}^{\pm }-1)$$

which implies the following decoders for the ON/OFF population still give us *g*(*x*) = *x*

(67) $$\phi_{i}^{\pm }=e_{i}\frac{4\sqrt{2}}{NM\pi }+e_{i}\frac{64\sqrt{2}}{5M\pi N}(1+a_{i}^{\pm })(4a^{\pm }{}_{i}^{2}-2a_{i}^{\pm }-1)$$

which yields the weight matrix

(68) $$\begin{array}{l} \omega_{ij}=\alpha_{i}\phi_{j}=e_{i}e_{j}\frac{4\sqrt{2}M}{N\pi }+e_{i}e_{j}\frac{64M\sqrt{2}}{5\pi N}(1+a_{j})\\ \;(4a_{j}^{2}-2a_{j}-1) \end{array}$$

Additionally, we can exploit symmetry to derive yet another weight matrix with precisely the same network of neurons:

(69) $$g^{+}(x)=\frac{1}{4}{\left(1+x\right)}^{2},\;g^{-}(x)=-\frac{1}{4}{\left(1-x\right)}^{2}$$

(70) $${\hat{P}}^{+}(a)=\frac{1}{M\pi }\sqrt{1+a},\;{\hat{P}}^{-}(a)=-\frac{1}{M\pi }\sqrt{1+a}$$

(71) $$\phi_{i}^{\pm }=\frac{\gamma (a_{i}^{\pm })}{N}=e_{i}\frac{2\sqrt{2}(1+a_{i}^{\pm })}{MN\pi }$$

(72) $$\omega_{ij}=e_{i}e_{j}\frac{2\sqrt{2}(1+a_{i}^{\pm })M}{N\pi }$$

and thus, we have the following three separate weight matrices

(73) $$\omega_{ij}=\alpha_{i}\phi_{j}=e_{i}e_{j}\frac{4\sqrt{2}M}{N\pi }$$

(74) $$\omega_{ij}=\alpha_{i}\phi_{j}=e_{i}e_{j}\frac{4\sqrt{2}M}{N\pi }+e_{i}e_{j}\frac{64M\sqrt{2}}{5\pi N}(1+a_{j})(4a_{j}^{2}-2a_{j}-1)$$

(75) $$\omega_{ij}=e_{i}e_{j}\frac{2\sqrt{2}(1+a_{j})M}{N\pi }$$

for *i, j* = 1, 2, …*N* that yield identical macroscopic dynamics from the same network of neurons as *N* → ∞. Furthermore, while all the weights converge to 0 as *N* → ∞, the scaled weights *Nω*_*ij*_ do not converge toward one another in the same limit. One important point is that none of the weights necessarily satisfy the constraint that the action of neuron *j* on all its downstream targets is the same, either excitatory or inhibitory. Or more precisely that

(76) $$\text{sign }\omega_{ij}=\text{sign }\omega_{i^{\prime }j}$$

for all *i, i*′, *j* = 1, 2, …*N*. Fortunately however, this issue has already been dealt with in the existing literature (Parisien et al., 2008). To summarize, one is able to take the weights generated by the NEF solution, and linearly transform them to yield a new network consisting of excitatory and inhibitory neurons (instead of ON/OFF) with weights that respect this constraint, which is related to Dale's principle.

**Figure 5:**
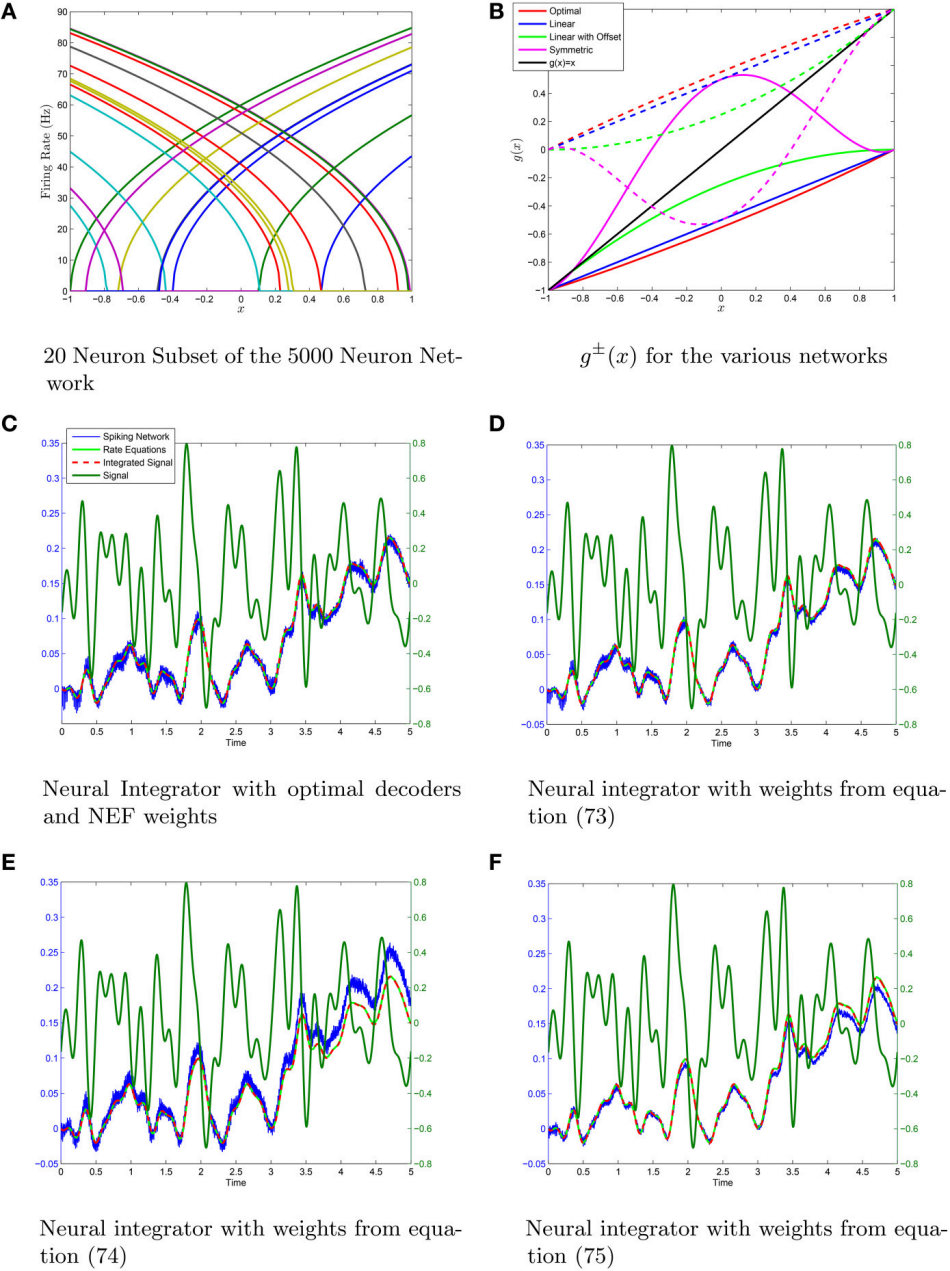
**Neural integrators generated using the same initial heterogeneous network of *N* = 5·10^3^ Theta neurons. (A)** A subset of 20 tuning curves from the network. **(B)** The different neural integrators are generated by using different *g*^±^(*x*) to form *g*(*x*). **(C–F)** The integrators generated using optimal decoders and decoders from Equations (63),(66), and (71) which results in weight matrcies Equations (73),(74), and (75). The scale-invariant decoders are fine-tuned with conjugate gradient descent. In all cases the fine-tuned decoders are very highly correlated (*p* > 0.98) with the scale-invariant decoders indicating only small perturbations off the scale-invariant decoder surface with substantial improvements in the root mean-squared error (RMSE) in computing ĝ(*z*) = *z*. The RMSE was *O*(10^−5^) with conjugate-gradient descent vs *O*(10^−2^) without. The variable λ = 0.01 was taken in the conjugate gradient descent fine-tuning.

**Figure 6:**
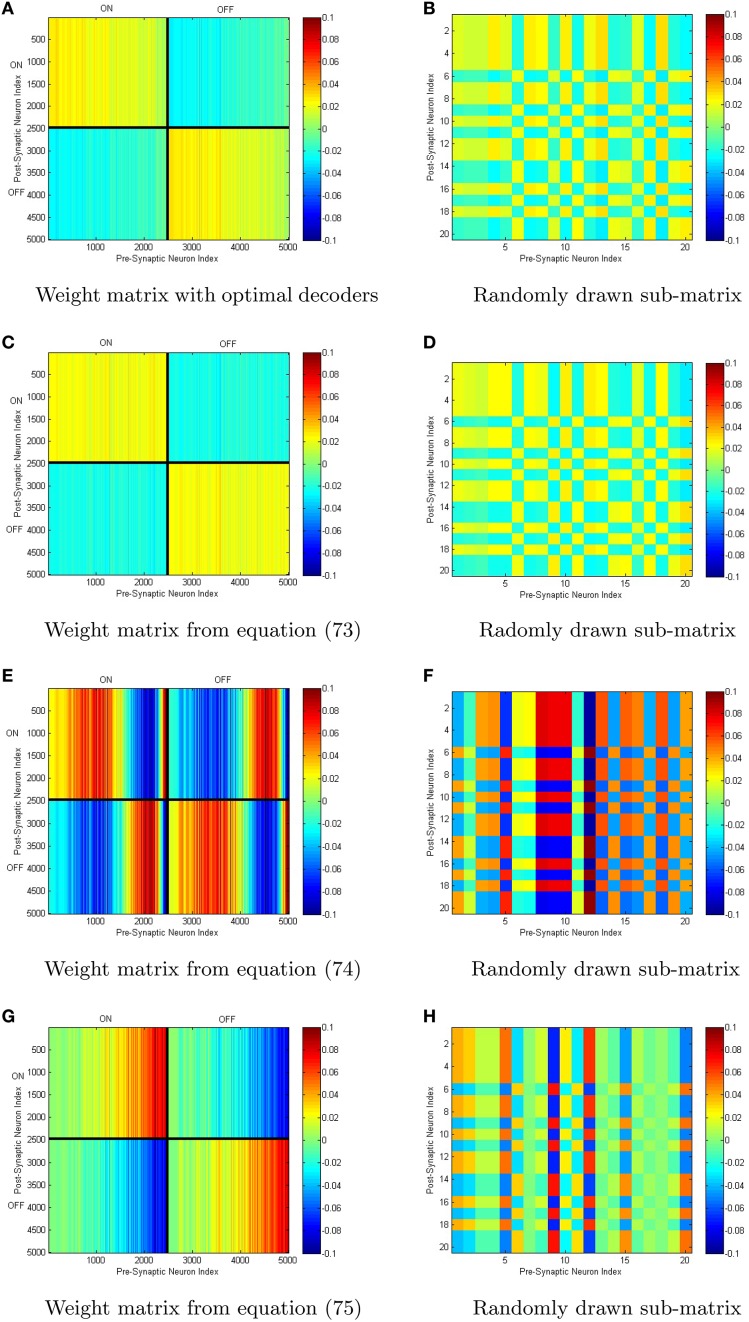
**(A–H)** Different connectivity weights can generate identical macroscopic dynamics from identical neuronal populations. Shown above are the weight matrices generated for the neural integrators in Figure 5. On the right are smaller sub-matrices of weights between 20 randomly selected neurons (the same 20 in each case).

We have simualted four neural networks with 5000 neurons each all generated with the same random sample drawn from the distribution (Equation 58) using the weights given by Equations (73–75), in addition to the weights generated by determining the optimal decoders. The scale-invariant decoders that correspond to Equations (73–75) were put through conjugate gradient fine-tuning with the final decoders being correlated to the initial decoders with a correlation coefficient greather than 0.98 in all cases. This lowered the root-mean-squared-error by 2–3 orders of magnitude with only slight perturbations off the scale-invariant decoding surface in each case. A sample set of tuning curves is shown in Figure 5A with the ĝ^±^(*x*) that correspond to the four different weight structures in Figure 5B. The neural integrators are shown in Figures 5C–F. The synaptic weights that correspond to the integrators are shown in Figure 6. The neurons have been sorted into ON/OFF populations and increasing *a* within a sub-population prior to plotting the weight matrix in the left column of Figure 6. A sub-sample of 20 neurons is also selected (which is identical across the four integrator networks) and their weights are plotted in the right column of Figure 6. For the optimal decoders, *g*^±^(*x*) ≈ ±(1 ± *x*)/2, which results in a weight structure similar to Equation (73). The weights differ substantially however in comparison to Equations (74) and (75) as the ĝ^±^ differ substantially from ±(1 ± *x*)/2

This example illustrates that identical networks of neurons can have identical macroscopic dynamics with vastly different weight matrices. While this is not particularly surprising as going from a microscopic description (the individual weights) to a macroscopic description (the dynamics) of a dynamical system is seldom a unique process, the surprising thing is one can explore this issue analytically. An important point to note is that even though the weight matrices are non-unique, they all have the form

(77) $$\omega_{ij}=f(e_{i},e_{j},a_{i})$$

and the weight matrices are nothing more than sample points drawn from different surfaces.

### 3.2. Exampe 2: Van der Pol oscillator

The Van der Pol oscillator (Van der Pol, 1926) is given by the dynamical system:

$$\dot{x}=\mu \left(x-\frac{1}{3}x^{3}-y\right)=F(x,y)$$

(78) $$\dot{y}=\frac{x}{\mu }=G(x)$$

Here, we will simulate the oscillator with large networks of neurons using the scale-invariant decoders with conjugate gradient descent fine tuning. As the decoding is linear, then from the above equations we only require the decoders for the functions *f*(*x, y*) = *x*, *f*(*x, y*) = *y* and *f*(*x, y*) = *x*^3^. We use a two-dimensional spherical coordinate system for the encoding ***e***_*i*_ = (cos(θ_*i*_), sin(θ_*i*_)) and assume uniform distributions in the *a*_*i*_ and the θ_*i*_. Note that:

(79) $$\begin{array}{l} x=\int_{0}^{2\pi }\int_{-1}^{\cos(\theta )x+\sin(\theta )y}\frac{\cos(\theta )}{\pi }\frac{2}{\pi \sqrt{1+a}}\\ \;\sqrt{\cos(\theta )x+\sin(\theta )y-\;a}dad\theta \\ y=\int_{0}^{2\pi }\int_{-1}^{\cos(\theta )x+\sin(\theta )y}\frac{\sin(\theta )}{\pi }\frac{2}{\pi \sqrt{1+a}}\\ \;\sqrt{\cos(\theta )x+\sin(\theta )y-\;a}dad\theta \\ x^{3}+3x=\int_{0}^{2\pi }\int_{-1}^{\cos(\theta )x+\sin(\theta )y}\frac{\cos(3\theta )+\cos(\theta )}{\pi }\frac{16{\left(\sqrt{1+a}\right)}^{3}}{\pi }\\ \;\sqrt{\cos(\theta )x+\sin(\theta )y-\;a}dad\theta \end{array}$$

which, immediately allows us to use the following scale-invariant decoders for the sub-functions *x, y*, and *x*^3^:

(80) $$\phi_{i}^{x}=\frac{4\sqrt{2}\cos\theta_{i}}{MN\pi }$$

$$\phi_{i}^{y}=\frac{4\sqrt{2}\sin\theta_{i}}{MN\pi }$$

(81) $$\phi_{i}^{x^{3}}=32\sqrt{2}\left(\cos(3\theta_{i})+\cos(\theta_{i})\right)\frac{{\left(1+a_{i}\right)}^{2}}{\pi MN}-3\phi_{i}^{x}$$

which yields the decoders for *F*(*x, y*) and *G*(*x, y*):

(82) $$\phi_{i}^{F}=\phi_{i}^{x}+\tau_{s}\mu \left(\phi^{x}-\frac{1}{3}\phi^{x^{3}}-\phi^{y}\right)$$

(83) $$\phi_{i}^{G}=\phi_{i}^{y}+\tau_{s}\frac{\phi^{x}}{\mu }$$

Thus, the weights are given by

(84) $$\omega_{ij}=\omega (\theta_{i},\theta_{j},a_{j})=M^{2}\cos(\theta_{i})\phi_{j}^{F}+M^{2}\sin(\theta_{i})\phi_{j}^{G}$$

**Figure 7:**
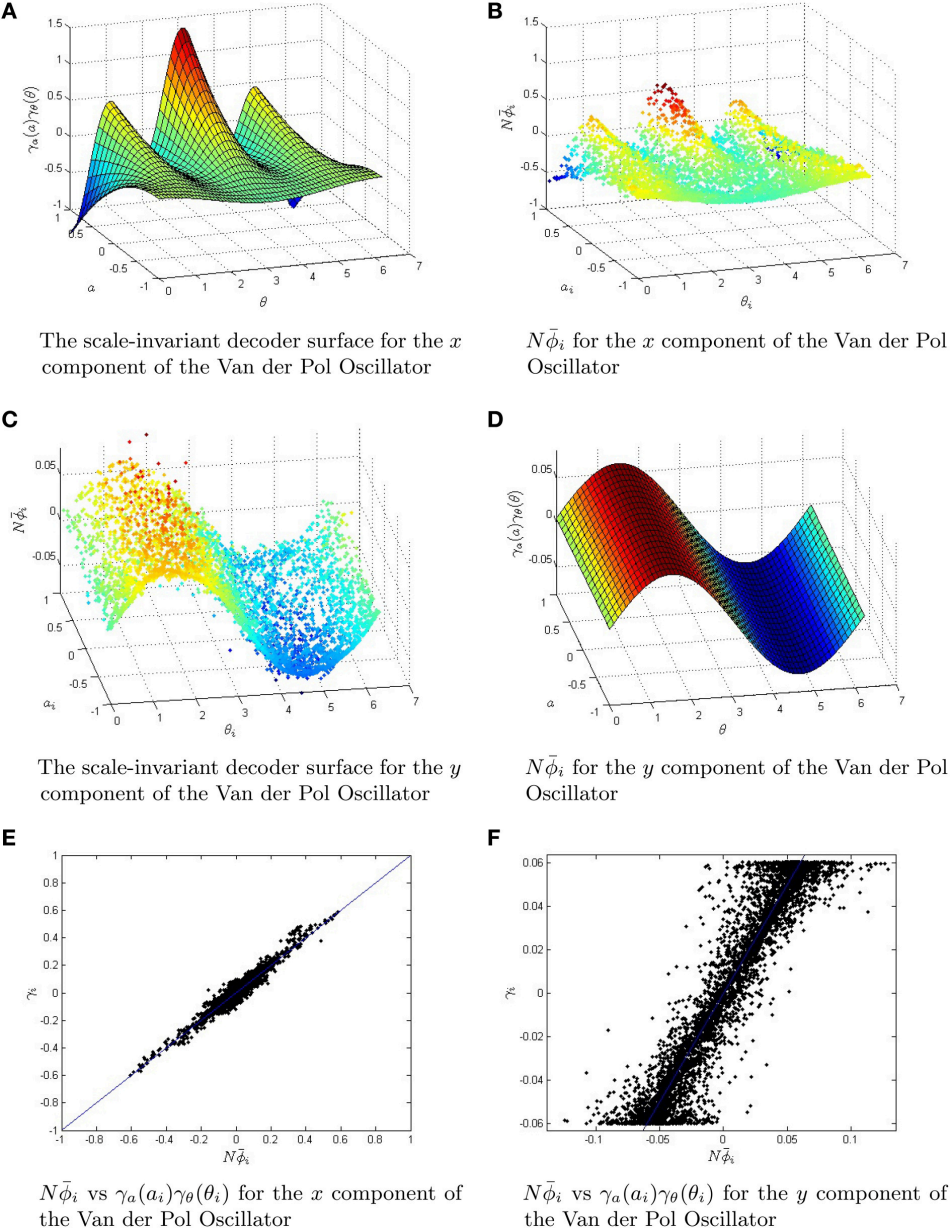
**The Van der Pol Oscillator is approximated by using a scale-invariant decoder surface**. The two functions *F*(*x, y*) and *G*(*x, y*) that are responsible for the dynamics of the Van der Pol Oscillator have scale-invariant decoder surfaces given by Equations (82,83). **(A,B)** The equations for the scale-invariant decoder surfaces are plotted in 3D. These surfaces are used to initialize conjugate gradient descent fine-tuning for a network of 5000 neurons. **(C,D)** The fine-tuned decoders $N{\bar{\phi }}_{i}$. They are slightly perturbed off of the surfaces in **(A,B)**. **(E,F)** The strong linear relationship in the scale-invariant surface and the conjugate gradient descent optimized decoders $N{\bar{\phi }}_{i}$. The correlation coefficient *r*≥0.95 in both cases, while the root mean squared error was reduced from *O*(10^−2^) to *O*(10^−5^). The parameter μ for the Van der Pol Oscillator was taken to be 0.7.

**Figure 8:**
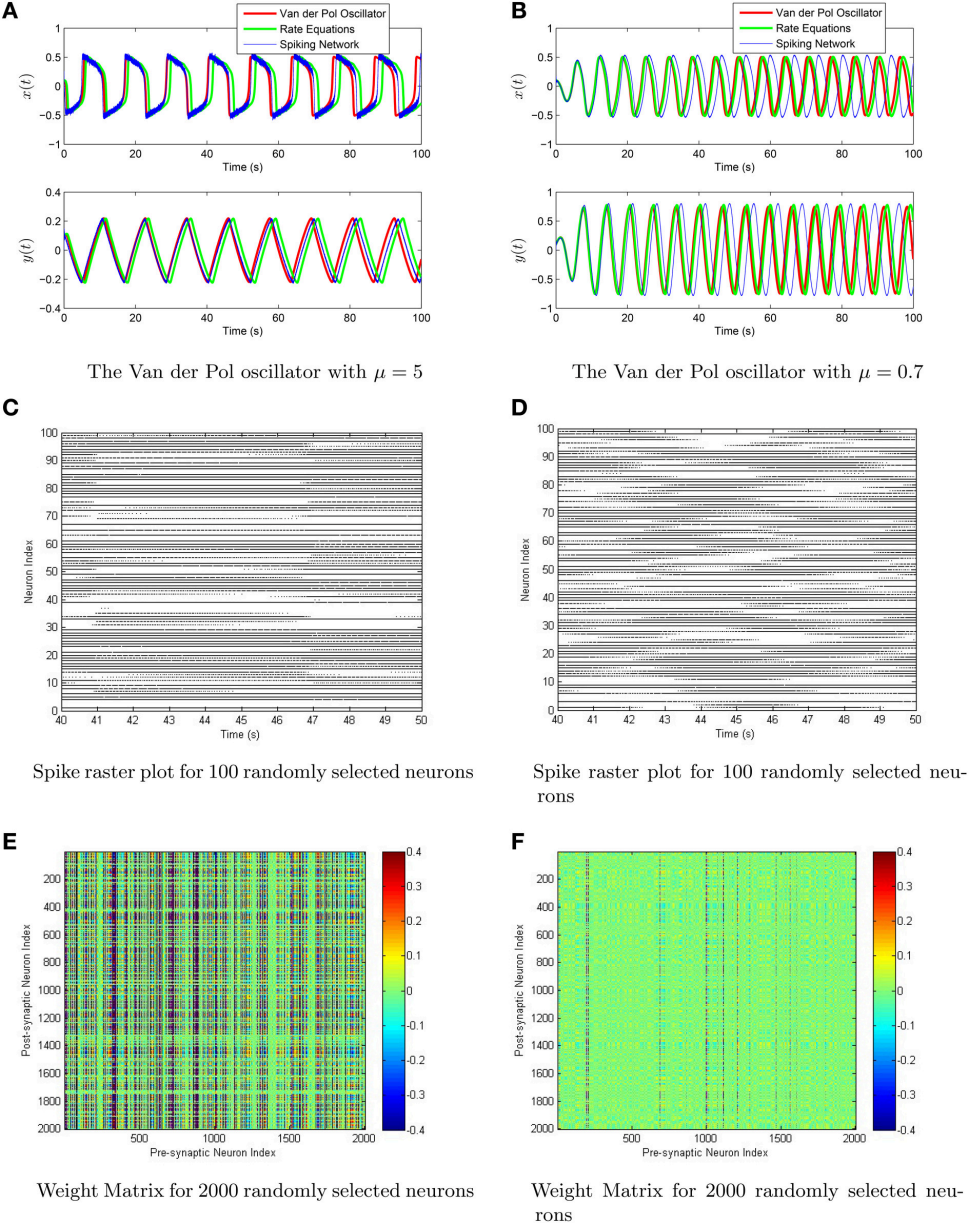
**(A,B)** The Van der Pol oscillator is simulated using a network of 10^5^ theta neurons with scale-invariant decoders after conjugate-gradient descent fine tuning in the relaxation oscillator regime (μ = 5, left column) and in the harmonic oscillator regime (μ = 0.7, right column). Shown in the top row is the comparison between the oscillator (red), the rate equations (green), and the spiking network (blue). **(C,D)** Shown in the middle is the spike raster plot for a 10 s interval of both networks. **(E,F)** The last row consists of a computed weight matrix for 2000 randomly selected neurons in the network.

Both the rate equations and the spiking neural network are simulated using the scale invariant decoders to weight the firing rates/spikes. To more explicitly show the effects of the conjugate-gradient descent fine tuning, we have plotted the scale-invariant decoder surfaces *Nϕ*^*F*^ and *Nϕ*^*G*^ in Figure 7, in addition to the decoders after conjugate gradient descent fine tuning. The scale-invariant decoders and the conjugate gradient descent fine-tuned decoders are again very tightly correlated with *r* > 0.95. We have simulated the Van der Pol Oscillator, as shown in Figure 8 in both the relaxation (μ = 5) and harmonic (μ = 0.7) oscillator regimes. In both cases, we have excellent correspondence with the network and the actual oscillator. The synaptic weight matrices are also shown in Figure 8 for a subset of neurons. Like the neural integrator, the weights again lie on a surface, only the surface is 3-dimensional.

### 3.3. Example 3: Lorenz attractor

The Lorenz system is given by the equations

(85) $$\dot{x}=\sigma (y-x)=F(x,y)$$

(86) $$\dot{y}=x(\rho -z)-y=G(x,y,z)$$

(87) $$\dot{z}=xy-\beta z=H(x,y,z)$$

and is known to exhibit chaotic behavior for specific values of σ, ρ and β (Lorenz, 1963). In order to approximate the Lorenz system, we require the decoders for the functions *x, y, z, xz* and *xy*. With a 3-dimensional spherical coordinate system, the encoding vectors ***e*** are given by ***e*** = (sin(θ)cos(ψ), sin(θ)sin(ψ), cos(θ)) where θ ∈ [0, π] ψ ∈ [0, 2π]. The decoders as a function of (ψ, θ, *a*) are given by:

(88) $$\begin{array}{l} \phi_{i}^{x}=\frac{4\sqrt{2}\cos(\psi_{i})}{NM\pi }\\ \phi_{i}^{y}=\frac{4\sqrt{2}\sin(\psi_{i})}{NM\pi }\\ \phi_{i}^{z}=\frac{8\sqrt{2}\cos(\theta_{i})}{NM\pi }\\ \phi_{i}^{xz}=\frac{24\sqrt{2}\cos(\theta_{i}-\psi_{i})(1+a_{i})}{2NM}-\frac{3\pi }{4}\phi_{i}^{y}\\ \phi_{i}^{xy}=\frac{64\sqrt{2}\sin(2\psi )(1+a_{i})}{NM\pi } \end{array}$$

which yields the decoders for *F, G, H* as:

(89) $$\phi_{i}^{F}=\sigma (\phi_{i}^{y}-\phi_{i}^{x})$$

$$\phi_{i}^{G}=\phi_{i}^{x}\rho -\phi_{i}^{xz}-\phi_{i}^{y}$$

(90) $$\phi_{i}^{H}=\phi_{i}^{xy}-\beta \phi_{i}^{z}$$

**Figure 9:**
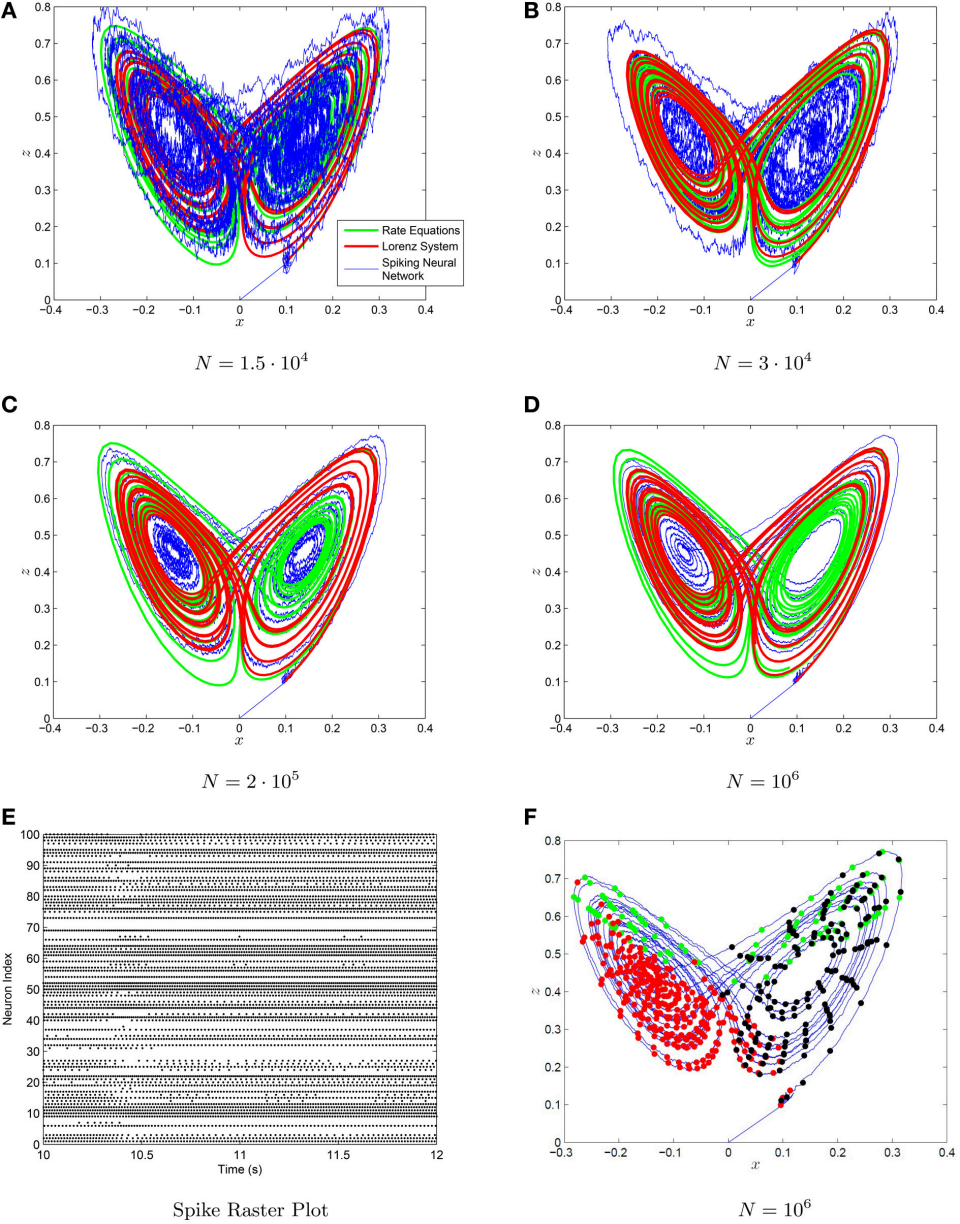
**(A–D)** The Lorenz attractor generated with a spiking neuronal network (blue) the neural rate equations (green) and integrating the Lorenz system (red) with neural networks of increasing size in figures. The decoders used are scale-invariant with conjugate gradient descent fine-tuning. The synaptic time constant was τ_*s*_ = 50 ms. **(E)** The spike raster plot for 100 neurons chosen at random from the *N* = 10^6^ neuron simulation. **(F)** The location of the spikes with regards to the strange attractor for three randomly selected neurons are shown in **(F)**.

The strange attractor generated by the Lorenz system and the neural rate equations using the decoders from Equations (88–90) are shown in Figure 9. The chaotic behavior and the strange attractor is also preserved when one uses a spiking neuronal network with the decoder weights on the spikes. Note that a great many neurons are required to adequately visualize the strange attractor, however the chaotic behavior is present even for smaller networks. We have also plotted the location of neural spiking with regards to the strange attractor. The neurons tend to spike more in specific regions of the strange attractor in accordance with their preferred orientation vectors and their *a*_*i*_ parameters. The weights are again given by the NEF formula:

(91) $$\begin{array}{l} \omega_{ij}=\omega (\theta_{i},\theta_{j},\psi_{i},\psi_{j},a_{j})=M^{2}\sin(\theta_{i})\cos(\psi_{i})\phi_{j}^{F}\\ \;+\;M^{2}\sin(\theta_{i})\sin(\psi_{i})\phi_{j}^{G}+M^{2}\cos(\theta_{i})\phi_{j}^{H} \end{array}$$

which defines a five dimensional surface.

## 4. Discussion

The NEF has been used to develop a wide variety of neural circuit models. The spiking networks generated from the NEF approach are spiking neural networks that are capable of functionally reproducing very sophisticated behaviors (Eliasmith, 2005). In the NEF approach, a synaptic weight between two neurons is a dot product of the post-synaptic neuron's preferred direction vector and the presynaptic neuron's linear decoding vector or “decoder.” The decoders weight the tuning curves for the neurons and are determined by an optimization criterion that minimizes the *L*_2_ error in the linear combination of tuning curves and the target dynamics of the network in addition to a factor that punishes the size of the decoders. The optimal decoders are unique, as they are determined by a convex optimization problem.

The first main point of this study is that in the large network limit, one can define a scale-invariant decoder that scales in inverse proportion to the network size *N* such that the scale-invariant decoders zero the cost function used to define the optimal decoders asymptotically in the mean-squared error. In the asymptotic limit, the scale-invariant decoders multiplied by the probability density governing the source(s) of heterogeneity converge to a constant function. The resulting linear combination of weighted firing rates converges to the tuning curve transforms asymptotically. We have determined this to also be the case with optimal decoders given by large matrix inversion, where the product of the optimal decoders and the heterogeneous density also converges to the weighted decoder. Furthermore, we have shown how one can invert tuning curve transform operators for type-I (theta model) and type-II firing rates. It turns out that the inversion is non-unique, which results in an infinite-dimensional family of scale-invariant decoder surfaces and thus synaptic weights.

The second contribution of this paper is a method of finding the scale-invariant decoders analytically from the decoded function and the probability density of the neuronal parameters. Additionally, we have demonstrated that as the weighted decoder is non-unique for any particular dynamics, the NEF solution to the weights with scale-invariant decoders also becomes non-unique.

Finally, we have demonstrated how the weights that couple neurons together can actually be thought of as surfaces defined entirely by the source(s) of heterogeneity in the network. In particular, a weight ω_*ij*_ is defined as the inner product of a scale-invariant decoder γ(***e***_*j*_, *a*_*j*_)/*N* of the post-synaptic neuron *j* with the encoding vector ***e***_*i*_ of the presynaptic neuron *i*. Thus, one can consider this inner product as a function over a higher dimensional space and the weights are merely sample points on this hypersurface. Furthermore, the weights generated via this approach have the advantage that the coupling between the *j*th presynaptic neuron and the *i*th postsynaptic neuron depends only upon the local properties of neuron *i* and neuron *j* for any specified dynamics. This is different from Hebbian plasticity, in that the coupling does not depend on presynaptic and postsynaptic activity, but rather on an intrinsic property of the presynaptic neuron times an intrinsic property of the postsynaptic neuron. While this perspective has been used before in the literature, for example to model hypercolumns in the primary visual cortex in (Shriki et al., 2006), here we show how it can be extended to arbitrary (smooth) dynamics.

### 4.1. Relationships between the analytical and optimal decoders

The optimal decoders appear to have an asymptotic weighted decoder, the product of the density function multiplied by the scaled optimal decoder, *NΦ*_*i*_, when one computes this quantity numerically after large-matrix inversion (see Figure 2). For the optimal decoders, the weighted decoder has high frequency oscillations that are related to the idiosyncracies of the particular sample of random neurons generated. These seem to attenuate with increasing network size, and regularization parameter λ. These high frequency oscillations are for example eliminated when the neurons are drawn from a grid. Indeed, when the neurons are drawn from a grid, the optimal decoders for a much larger network can be approximated by simply interpolating between the decoder values for the smaller grid network, and rescaling the interpolated decoders in accordance with the network size.

Thus, one may ask is the weighted decoder generated by the optimal decoders (1) convergent as *N* → ∞, and (2) does it converge to any particular weighted decoder in the set defined by the requirement that ĝ(*x*) = *M*(*P*^+^, *P*^−^) = *L*^+^(*P*^+^) + *L*^−^(*P*^−^) = *g*(*x*)? It seems that numerically the quantity *Nρ*_*a*_(*a*_*i*_)Φ_*i*_ does converge to some surface *P*^±^ that varies depending on the identity of neuron *i* as an ON/OFF neuron. The likely candidate for the specific ${\hat{P}}^{\pm }$ in the set defined by *M*(*P*^+^, *P*^−^) is the surface that minimizes Equation (42). However, the relationship between the optimal decoders, and any particular scale-invariant decoder as *N* → ∞ is outside of the scope of this paper and is best left for future work. Relatedly, there seem to be some systematic differences between scale-invariant and optimal decoders due to finite neurons. For example, a scale-invariant decoder surface can have discontinuities, while corresponding regularized optimal decoder surfaces do not, even with very large numbers of neurons

### 4.2. Relationship to other approaches

We have demonstrated that if one defines a scale-invariant decoder that is a function of the source(s) of heterogeneity of the neurons, one can obtain arbitrary macroscopic dynamics with the NEF weight solution, which is the dot product of the decoder for the presynaptic neuron, and the preferred orientation vector or encoding vector for the post-synaptic neuron. Thus, both the weights and the decoders for the neurons can be thought of as surfaces as they are merely functions of the sources of heterogeneity of the network. While this perspective is novel in the sense that we have shown one can obtain arbitrary dynamics with this approach, similar decoders and weights have been suggested in previous work. In particular, the scale-invariant decoders here can be thought of as an extension of the neuronal population vectors of Georgopolous (Georgopoulos et al., 1986, 1994). It has been previously noted (for example in Eliasmith and Anderson, 2004) that the population vector from (Georgopoulos et al., 1986, 1994) is similar to the case where one uses the encoding vectors *e*_*i*_ as the decoding vectors ϕ_*i*_. Here, we have derived a more general approach where ϕ_*i*_ is a function of ***e***_*i*_ and the other sources of heterogeneity in the tuning curves. Additionally, weights that are a function of the presynaptic and post-synaptic preferred orientations have been also used in the literature (Ben-Yishai et al., 1995; Shriki et al., 2006).

There are also methods in the literature that construct networks of neurons with prescribed dynamics. For example, spiking networks of leaky integrate-and-fire models have been constructed that can display arbitrary linear dynamics with weights that are seemingly unrelated to the NEF solution for the synaptic weights (Boerlin et al., 2013). The solution obtained in (Boerlin et al., 2013) is derived through minimizing the time integral of the L2 norm of the decoded estimate of the dynamics, $\hat{x}\left(t\right)$ and the intended dynamics in addition to other terms intended to minimize the spiking, and distribute the spikes equitably across the network. The end result is a network of leaky integrate and fire neurons and weights that when coupled together display the desired dynamics. While the solution is very elegant, it is unknown if it can be extended to non-linear dynamics.

### 4.3. Numerical applications

We considered whether this work has practical applications for neural simulations. As an example, we considered whether (having found the weighted decoders for a given network) it would be useful to adjust ρ in order to make the weights uniform, thus avoiding large fluctuations associated with the spiking of heavily weighted neurons. This sometimes led to modest reductions in spike noise in simulations.

As discussed above, scale-invariant decoders can serve as a starting point for iterative optimization, quickly leading to highly optimized weights for large networks. However, it should be pointed out that since the optimization problem is convex, any starting point can be used, and in our experience scale-invariant decoders are only moderately better than other reasonable choices.

It is possible that our approach could be applied to groups of neurons that are so large as to be impractical even for efficient iterative methods. The cortex consists of a fairly continuous sheet of neurons with few distinct boundaries, suggesting that it may be somehow useful to simultaneously consider the activity of billions of neurons. On the other hand, if the goal is simply to optimize synaptic weights, the degree of convergence onto single neurons (< 200, 000) is within a practical range for iterative methods. Furthermore, our solution for multivariable functions requires encoder distributions that are separable in hyper-spherical coordinates, which may be a limitation for modeling extensive sheets of neurons with overlapping tuning.

In light of these experiences, we consider this work to be valuable mainly as a source of new insights into network function and dynamics, rather than as a basis for new numerical tools.

### 4.4. Analyzing measured synaptic weights

Using experimental methods, it is possible to measure both the synaptic weights (defined as the peak post-synaptic current) in addition to fitting integrate-and-fire type neurons with heterogeneity using the dynamic current-voltage curve approach (Badel et al., 2008; Harrison, 2014; Harrison et al., 2015). While there are typically many parameters for more complicated integrate-and-fire models (such as the AdEx), one can always reduce the number of heterogeneous parameters to a much smaller set governing the properties of the tuning curves. We refer to these generically as ***b***_*i*_, a vector with the parameter values for the *i*th neuron. Given these assumptions, and the work done in this paper, one kind of analysis that can be conducted without much effort is the construction of a non-linear regression model of the weights:

(92) $$\omega_{ij}=F(b_{i},b_{j})+\epsilon_{ij}$$

where *F*(***b***_*i*_, ***b***_*j*_) is either a non-linear or linear model with a number of free coefficients and ω_*ij*_ are the experimentally determined synaptic weights of a recurrently coupled neural network. The coefficients can be estimated using optimization techniques to minimize ϵ_*ij*_. For this weight analysis to be valid, we require the following

$$|\epsilon_{ij}|\ll |F(b_{i},b_{j})|,\forall i,j$$

where $F\left(b_{i},b_{j}\right)=O\left(N^{-1}\right)$. The core result of this paper is that any dynamics are possible with synaptic weights of the form (Equation 92), and their is no unique weight matrix that confers specfic dynamics. Thus, a regression analysis of the synaptic weights (Equation 92) is a reasonable analysis to conduct if one knows the sources of heterogeneity for the neurons in the network and provided that the residuals are sufficiently small.

### 4.5. Future work

While the networks constructed here display the desired macroscopic dynamics, this is not always the case. In particular, if the time constants are too small, then the collective macroscopic state can destabilize. For the weight solution we have determined to be valid, one needs to prove that the macroscopic dynamics form a stable attractor in the large network limit. Unfortunately, resolution of this problem is well outside the scope of this paper. While a great deal of work has been done in determing the stability of asynchronous states in large network limits (for example in Abbott and van Vreeswijk, 1993; van Vreeswijk, 1996), to our knowledge no work has been when the weights have structure present here. The majority of work done on large network stability analysis is devoted to weights that are constant throughout the network, ω_*ij*_ = ω however there is some work on non-constant, randomly distributed weights (Hermann and Touboul, 2012). The authors of (Hermann and Touboul, 2012) note that oscillations arise when considering randomly distributed weights. Here, we demonstrate that with a little bit more structure to the weights/network (the weights are functions of the properties of the *f*(*I*) curves of the neurons), arbitrary prescribed macroscopic dynamics can be generated by the network.

Networks of heterogeneous theta oscillators have been extensively analyzed in (Barreto et al., 2008; Luke et al., 2013; So et al., 2014) by using the Ott-Antonsen Anzats initially applied to networks of Kuramoto Oscillators (Martens et al., 2009; Ott and Antonsen, 2009). Additionally, one of the weight solutions for a network with one-dimensional dynamics that arises from the scale-invariant decoders sets all the weights to ±ω by setting the density to $\rho \propto |\hat{P}\left(a\right)|$ where the constant of proportionality normalizes $\left|\hat{P}\left(a\right)\right|$. Given that, it may be possible to apply some of the existing literature on the stability analysis of networks of heterogeneous theta neurons to this network.

In addition to stability analysis of the large network, the weight solutions were derived here under a pair of simplifying assumptions. In particular the two strongest assumptions were that the FI curves were constant in time, and that the neurons were coupled using current-based synapses instead of conductance based synapses. We intend to extend the approach we have taken here with scale-invariant decoders to neurons with conductance based synapses, and *f*(*I*) curves that vary due to forces like spike frequency adaptation (Ermentrout, 2006). Fortunately, some of the initial work on generating macroscopic rate-equations (a necessary initial step) for conductance based neurons has been done in (Ermentrout, 1994; Shriki et al., 2006), in addition to work on rate equations for adapting neurons (Nicola and Campbell, 2013b)

## Author contributions

WN and MS performed much of the analysis. The numerical experiments were performed by WN with assistance from BT with regards to the Neural Engineering Framework. WN, BT, and MS contributed to the writing of the manuscript.

## Funding

This research was funded by an NSERC Postgraduate Scholarships-Doctoral Program (WN) and NSERC discovery grants (BT, MS).

### Conflict of interest statement

The authors declare that the research was conducted in the absence of any commercial or financial relationships that could be construed as a potential conflict of interest.

## Appendix

### Determining the decoder surface

Here, we will make use of the Laplace transform to analytically determine the functions one can represent and numerically invert for the decoder surfaces in the NEF approach. In particular, consider the operator $L^{+}\left(\hat{P}\right)$ which is given by

(A1) $${\hat{g}}^{+}(x)=L^{+}(\hat{P})=\int_{-1}^{x}\hat{P}(a)\sqrt{x-a}\;da$$

Using a series of substitutions we can write the following:

(A2) $${\hat{g}}^{+}(x)=\int_{0}^{x+1}\hat{P}(b-1)\sqrt{x+1-b}\;db$$

(A3) $$\begin{array}{l} \;=\int_{0}^{y}\hat{P}(b-1)\sqrt{y-b}\;db\\ \;=\int_{0}^{y}Q(b)R(y-b)\;db=m(y) \end{array}$$

where $Q\left(b\right)=\hat{P}\left(b-1\right)=\hat{P}\left(a\right)$ and $R\left(y-b\right)=\sqrt{y-b}=\sqrt{x+1-b}=\sqrt{x-a}$, and *m*(*y*) = ĝ(*y*−1) = ĝ(*x*). In this case, the expectation is a convolution when written in the appropriate variables:

(A4) m(z)=(Q⋆R)(z)

where ⋆ denotes the convolution operator. Taking a Laplace transform yields:

(A5) $$ℒ(m(z))=ℒ(Q(z))ℒ(R(z))=ℒ(Q(z))\frac{\sqrt{\pi }}{2s^{3/2}}$$

(A6) $$\Rightarrow ℒ(Q(z))=\frac{2}{\sqrt{\pi }}ℒ(m(z))s^{3/2}$$

(A7) =ℒ(m(z))ℒ(C(z))

for some function *C*(*z*). Unfortunately $ℒ(C(z))=\frac{2}{\sqrt{\pi }}s^{3/2}$ is not invertible as we have:

(A8) $$ℒ(\sqrt{t})=\frac{1}{2}\frac{\sqrt{\pi }}{s^{3/2}}\;ℒ(f^{\prime \prime }(t))=s^{2}ℒ(f(t))+sf(0)+f^{\prime }(0)$$

from the general properties of the Laplace transform. This implies that if $f\left(t\right)=-4\sqrt{t}$, *f*″(*t*) = *t*^−3/2^ and the Laplace transform is undefined as *f*′(0) → ∞. Suppose instead that we assume that *m*(*z*) is twice differentiable and that *m*(0) = 0 and *m*′(0) = 0. Then we can write the following:

(A9) $$ℒ(Q(z))=\frac{2}{\sqrt{\pi }}s^{3/2}ℒ(m(z))$$

(A10) $$\begin{array}{l} \;=\frac{2}{\sqrt{\pi }}s^{-1/2}ℒ(m^{\prime \prime }(z))\\ \;=ℒ\left(\frac{2}{\pi \sqrt{z}}\right)ℒ(m^{\prime \prime }(z))\\ \Rightarrow Q(z)=\int_{0}^{z}\frac{2}{\pi \sqrt{t}}m^{\prime \prime }(z-t)dt \end{array}$$

This is how to find *Q*(*z*) as a convolution with *m*″(*z*), the second derivative of the function you want to approximate and $1/\sqrt{t}$. Based on these assumptions, we require that *m*(*z*) = *bz*^2^ + *O*(*z*^3^). Writing the convolution in terms of the original variables, we have the following:

(A11) $$\hat{P}(a)=\int_{0}^{a+1}\frac{2}{\pi \sqrt{t}}{\hat{g}}^{\prime \prime }(a-t)dt$$

This implies that we can approximate any function that vanishes to two orders at *x* = −1 using the following decoder for the ON neurons:

(A12) $$\phi_{i}^{+}(a_{i})=\frac{2}{N}\frac{1}{\rho_{a}(a_{i})}\int_{0}^{a_{i}+1}\frac{2}{\pi \sqrt{t}}{\hat{g}}^{\prime \prime }(a_{i}-t)dt$$

Additionally, if one repeats this process for a population of OFF neurons, then we can immediately write down the decoder for a function *g* that vanishes to two orders at *x* = 1:

(A13) $$\phi_{i}^{-}(a_{i})=\frac{2}{N}\frac{1}{\rho_{a}(a_{i})}\int_{0}^{a_{i}+1}\frac{2}{\pi \sqrt{t}}{\hat{g}}^{\prime \prime }(-a_{i}-t)dt$$

Given the constraints on the derivatives of ĝ, it is clear that using a population of ON and OFF neurons, the resulting approximant has the form:

(A14) $$\begin{array}{l} \hat{g}(x)={\hat{g}}^{+}(x)+{\hat{g}}^{-}(x)\\ \;=\frac{1}{2}\left(g(x)-g(-1)-(x+1)g^{\prime }(-1)\right)\\ \;+\;\frac{1}{2}\left(g(x)-g(1)-(x-1)g^{\prime }(1)\right)\\ \;=g(x)-A-Bx \end{array}$$

by using the ON and OFF populations, where *g*(*x*) is the function we want to approximate. The remainder is a linear term, *A* + *Bx*. Thus, if we can approximate an arbitrary linear function with a population of type-1 neurons with heterogeneity, then we can accomodate the remainder term. Note the following:

(A15) $$\begin{array}{l} \frac{2}{\pi }\int_{-1}^{\pm x}\frac{\sqrt{\pm x-a}}{\sqrt{a+1}}\;da=\frac{4}{\pi }\int_{0}^{\sqrt{x+1}}\sqrt{x+1-u^{2}}du\\ \;=1\pm x \end{array}$$

Thus, with the functions ${\hat{P}}^{+}=\frac{2C}{\pi \sqrt{1+a}}$ and ${\hat{P}}^{-}=\frac{2D}{\pi \sqrt{1-a}}$, for the ON and OFF neurons respectively, we can approximate

C(x+1)+D(1−x)=(C−D)x+(C+D)=Bx+A

with *C* = (*A* + *B*)/2 and *D* = (*A* − *B*)/2. Thus, to approximate the function *g*(*x*), we can use the following $\hat{P}$:

(A16) $$\begin{array}{l} {\hat{P}}^{+}(a)=\frac{2}{\pi }\left(\frac{g(-1)+g(1)}{2}+g^{\prime }(-1)\right)\frac{1}{\sqrt{1+a}}\\ \;+\int_{0}^{a+1}g^{\prime \prime }(a-t)\frac{2}{\pi \sqrt{t}}dt \end{array}$$

(A17) $$\begin{array}{l} {\hat{P}}^{-}(a)=\frac{2}{\pi }\left(\frac{g(-1)+g(1)}{2}-g^{\prime }(1)\right)\frac{1}{\sqrt{1+a}}\\ \;+\int_{0}^{a+1}g^{\prime \prime }(-t-a)\frac{2}{\pi \sqrt{t}}dt \end{array}$$

To remove the linear error, we used a linear combination of 1 + *x* and 1 − *x*. This choice is not unique. For example, we could have used

(A18) $$g^{\pm }(x)=\frac{{\left(1\pm x\right)}^{2}}{1+x^{2}}$$

to eliminate constant term and a separate linear combination of

(A19) $$g^{\pm }(x)=1\pm x^{2}$$

to eliminate the *x* term. The reason Equations (A18, A19) are not used for type-I neurons is due to complexity in the associated weighted decoders. However, these forms are simpler for Type-II firing rates and hence they are used there.

The same process can be carried out with the type-II firing rate form, which yields the operators:

(A20) $$L^{\pm }({\hat{P}}^{\pm })=\int_{-1}^{\pm x}{\hat{P}}^{\pm }(a)(\pm x-a+c)\;da$$

with the resulting values for ${\hat{P}}^{\pm }$ being

(A21) $${\hat{P}}^{\pm }(a)=2A+2Ba\frac{\left(a^{2}-3\right)}{{\left(a^{2}+1\right)}^{3}}+g^{\prime \prime }(\pm a)$$

for *c* = 0. For *c* > 0, the inversion for functions that vanish to second order at *x* = ±1 yields

(A22) $${\hat{P}}^{\pm }(a)=\frac{1}{c}\int_{0}^{a+1}\exp\left(-\frac{t}{c}\right)g^{\prime \prime }(\pm (a-t))\;dt$$

One can use the convolution (A22) to compute the weighted decoders for

$${\hat{g}}^{\pm }(x)=\frac{{\left(1\pm x\right)}^{2}}{1+x^{2}}$$

the solution is lengthy and thus we do not include it here.

## References

[B1] Abbott L. F. van Vreeswijk C. (1993). Asynchronous states in networks of pulse-coupled oscillators. Learn. Mem. 48, 1483–1490. 9960738 10.1103/physreve.48.1483

[B2] Abbott L. F. (1999). Lapicques introduction of the integrate-and-fire model neuron (1907). Brain Res. Bull. 50, 303–304. 10643408 10.1016/s0361-9230(99)00161-6

[B3] Ahmed B. Anderson J. C. Douglas R. J. Martin K. A. Whitteridge D. (1998). Estimates of the net excitatory currents evoked by visual stimulation of identified neurons in cat visual cortex. Cereb. Cortex 8, 462–476. 9722089 10.1093/cercor/8.5.462

[B4] Azouz R. Gray C. M. Nowak L. G. McCormick D. A. (1997). Physiological properties of inhibitory interneurons in cat striate cortex. Cereb. Cortex 7, 534–545. 9276178 10.1093/cercor/7.6.534

[B5] Badel L. Lefort S. Brette R. Petersen C. C. H. Gerstner W. Richardson M. J. E. (2008). Dynamic iv curves are reliable predictors of naturalistic pyramidal-neuron voltage traces. J. Neurophysiol. 99, 656–666. 10.1152/jn.01107.2007 18057107

[B6] Barreto E. Hunt B. Ott E. So P. (2008). Synchronization in networks of networks: the onset of coherent collective behavior in systems of interacting populations of heterogeneous oscillators. Phys. Rev. E 77:036107. 10.1103/PhysRevE.77.036107 18517461 PMC2453534

[B7] Bekolay T. Laubach M. Eliasmith C. (2014). A spiking neural integrator model of the adaptive control of action by the medial prefrontal cortex. J. Neurosci. 34, 1892–1902. 10.1523/JNEUROSCI.2421-13.2014 24478368 PMC6827589

[B8] Ben-Yishai R. Bar-Or R. L. Sompolinsky H. (1995). Theory of orientation tuning in visual cortex. Proc. Natl. Acad. Sci. U.S.A. 92, 3844–3848. 7731993 10.1073/pnas.92.9.3844 PMC42058

[B9] Bishop C. M. (1995). Neural Networks for Pattern Recognition. Oxford, UK: Oxford University Press.

[B10] Bobier B. Stewart T. C. Eliasmith C. (2014). A unifying mechanistic model of selective attention in spiking neurons. PLoS Comput. Biol. 10:e1003577. 10.1371/journal.pcbi.1003577 24921249 PMC4055282

[B11] Boerlin M. Machens C. K. Denève S. (2013). Predictive coding of dynamical variables in balanced spiking networks. PLoS Comput. Biol. 9:e1003258. 10.1371/journal.pcbi.1003258 24244113 PMC3828152

[B12] Brette R. Gerstner W. (2005). Adaptive exponential integrate-and-fire model as an effective description of neuronal activity. J. Neurophysiol. 94, 3637–3642. 10.1152/jn.00686.2005 16014787

[B13] Brunel N. Van Rossum M. C. W. (2007). Lapicques 1907 paper: from frogs to integrate-and-fire. Biol. Cybern. 97, 337–339. 10.1007/s00422-007-0190-0 17968583

[B14] Conklin J. Eliasmith C. (2005). A controlled attractor network model of path integration in the rat. J. Comput. Neurosci. 18, 183–203. 10.1007/s10827-005-6558-z 15714269

[B15] Dayan P. Abbott L. F. (2001). Theoretical Neuroscience, Vol. 806. Cambridge, MA: MIT Press.

[B16] DeWolf T. Eliasmith C. (2011). The neural optimal control hierarchy for motor control. J. Neural Eng. 8:065009. 10.1088/1741-2560/8/6/065009 22056418

[B17] Eliasmith C. Anderson C. H. (2004). Neural Engineering: Computation, Representation, and Dynamics in Neurobiological Systems. MIT press.

[B18] Eliasmith C. Stewart T. C. Choo X. Bekolay T. DeWolf T. Tang C. Rasmussen D. (2012). A large-scale model of the functioning brain. Science 338, 1202–1205. 10.1126/science.1225266 23197532

[B19] Eliasmith C. (2005). A unified approach to building and controlling spiking attractor networks. Neural Comput. 17, 1276–1314. 10.1162/0899766053630332 15901399

[B20] Ermentrout G. B. Kopell N. (1986). Parabolic bursting in an excitable system coupled with a slow oscillation. SIAM J. Appl. Math. 46, 233–253.

[B21] Ermentrout G. B. (1994). Reduction of conductance-based models with slow synapses to neural nets. Neural Comput. 6, 679–695.

[B22] Ermentrout B. (2006). Linearization of f-i curves by adaptation. Neural Comput. 10, 1721–1729. 10.1162/089976698300017106 9744894

[B23] Georgopoulos A. P. Schwartz A. B. Kettner R. E. (1986). Neuronal population coding of movement direction. Science 233, 1416–1419. 3749885 10.1126/science.3749885

[B24] Georgopoulos A. P. Lurito J. T. Petrides M. Schwartz A. B. Massey J. T. (1994). Mental rotation of the neuronal population vector. Biol. Comput. Physicist. Choice 3, 183. 10.1126/science.2911737 2911737

[B25] Harrison P. M. Badel L. Wall M. J. Richardson M. J. E. (2015). Experimentally verified parameter sets for modelling heterogeneous neocortical pyramidal-cell populations. PLoS Comput. Biol. 11:e1004165. 10.1371/journal.pcbi.1004165 26291316 PMC4546387

[B26] Harrison P. M. (2014). Experimentally Verified Reduced Models of Neocortical Pyramidal Cells. Ph.D. thesis, University of Warwick.

[B27] Hermann G. Touboul J. (2012). Heterogeneous connections induce oscillations in large-scale networks. Phys. Rev. Lett. 109:018702. 10.1103/PhysRevLett.109.018702 23031137

[B28] Izhikevich E. M. (2003). Simple model of spiking neurons. Neural Netw. IEEE Trans. 14, 1569–1572. 10.1109/TNN.2003.820440 18244602

[B29] Izhikevich E. M. (2007). Dynamical Systems in Neuroscience. Cambridge, MA: MIT Press.

[B30] Lapicque L. (1907). Recherches quantitatives sur lexcitation électrique des nerfs traitée comme une polarisation. J. Physiol. Pathol. Gen. 9, 620–635.

[B31] Lorenz E. N. (1963). Deterministic nonperiodic flow. J. Atmos. Sci. 20, 130–141.

[B32] Luke T. B. Barreto E. So P. (2013). Complete classification of the macroscopic behavior of a heterogeneous network of theta neurons. Neural Comput. 25, 3207–3234. 10.1162/NECO_a_00525 24047318

[B33] Martens E. A. Barreto E. Strogatz S. H. Ott E. So P. Antonsen T. M. (2009). Exact results for the kuramoto model with a bimodal frequency distribution. Phys. Rev. E 79:026204. 10.1103/PhysRevE.79.026204 19391817

[B34] MATLAB (2014). version 7.10.0 (R2010a). Natick, MA: The MathWorks Inc.

[B35] Naud R. Marcille N. Clopath C. Gerstner W. (2008). Firing patterns in the adaptive exponential integrate-and-fire model. Biol. Cybern. 99, 335–347. 10.1007/s00422-008-0264-7 19011922 PMC2798047

[B36] Nesse W. Borisyuk A. Bressloff P. (2008). Fluctuation-driven rhythmogenesis in an excitatory neuronal network with slow adaptation J. Comput. Neurosci. 25, 317–333. 10.1126/science.1225266 18427966

[B37] Nicola W. Campbell S. A. (2013a). Bifurcations of large networks of two-dimensional integrate and fire neurons. J. Comput. Neurosci. 35, 87–108. 10.1007/s10827-013-0442-z 23430291

[B38] Nicola W. Campbell S. A. (2013b). Mean-field models for heterogeneous networks of two-dimensional integrate and fire neurons. Front. Comput. Neurosci. 7:184. 10.3389/fncom.2013.00184 24416013 PMC3873638

[B39] Ott E. Antonsen T. M. (2009). Long time evolution of phase oscillator systems. Chaos 19:023117. 10.1063/1.3136851 19566252

[B40] Parisien C. Anderson C. H. Eliasmith C. (2008). Solving the problem of negative synaptic weights in cortical models. Neural Comput. 20, 1473–1494. 10.1162/neco.2008.07-06-295 18254696

[B41] Rasmussen D. Eliasmith C. (2014). A spiking neural model applied to the study of human performance and cognitive decline on raven's advanced progressive matrices. Intelligence 42, 53–82. 10.1016/j.intell.2013.10.003

[B42] Salinas E. Abbott L. F. (1995). Vector reconstruction from firing rates. J. Comput. Neurosci. 1, 89–107. 8792227 10.1007/BF00962720

[B43] Shriki O. Hansel D. Sompolinsky H. (2006). Rate models for conductance-based cortical neuronal networks. Neural Comput. 15, 1809–1841. 10.1162/08997660360675053 14511514

[B44] Singh R. Eliasmith C. (2006). Higher-dimensional neurons explain the tuning and dynamics of working memory cells. J. Neurosci. 26, 3667–3678. 10.1523/JNEUROSCI.4864-05.2006 16597721 PMC6674128

[B45] So P. Luke T. B. Barreto E. (2014). Networks of theta neurons with time-varying excitability: Macroscopic chaos, multistability, and final-state uncertainty. Phys. D Nonlinear Phenomena 267, 16–26. 10.1016/j.physd.2013.04.009

[B46] Stafstrom C. E. Schwindt P. C. Crill W. E. (1984). Repetitive firing in layer v neurons from cat neocortex *in vitro.* J. Neurophysiol. 52, 264–277. 6481432 10.1152/jn.1984.52.2.264

[B47] Touboul J. (2008). Bifurcation analysis of a general class of nonlinear integrate-and-fire neurons. SIAM J. Appl. Math. 68, 1045–1079. 10.1137/070687268

[B48] Van der Pol B. (1926). Lxxxviii. on relaxation-oscillations. Lond. Edinburgh Dublin Philos. Mag. J. Sci. 2, 978–992.

[B49] van Vreeswijk C. V. (1996). Partial synchronization in populations of pulse-coupled oscillators. Phys. Rev. E 54, 5522. 9965739 10.1103/physreve.54.5522

[B50] Werfel J. Xie X. Seung H. S. (2005). Learning curves for stochastic gradient descent in linear feedforward networks. Neural Comput. 17, 2699–2718. 10.1162/089976605774320539 16212768

