# Supplementary material for: Obtaining Arbitrary Prescribed Mean Field Dynamics for Recurrently Coupled Networks of Type-I Spiking Neurons with Analytically Determined Weights
Source: Front Comput Neurosci. 2016 Feb 29;10:15. doi: 10.3389/fncom.2016.00015 (PMC4770054; doi:10.3389/fncom.2016.00015)
Supplement: Supplementary file 1 [file DataSheet1.PDF]

Supplementary Material: Obtaining Arbitrary Prescribed Mean Field Dynamics for Recurrently Coupled Networks of Type-I Spiking Neurons with Analytically Determined Weights

Wilten Nicola*, Bryan Tripp and Matthew Scott
*Correspondence:
Author Name: Wilten Nicola
wnicola@uwaterloo.ca

1 PIECEWISE SMOOTH CONTINUOUS FUNCTIONS

In this section, we will show how we can approximate the piecewise defined function

$$h(x) = \begin{cases} h_L(x) & x < b \\ h_R(x) & x > b \end{cases} \tag{1}$$

with scale-invariant decoders. Consider a population of ON neurons with the following weighted decoder:

$$\hat{P}(a) = \begin{cases} P_L(a) & a < 0 \\ P_R(a) & a > 0 \end{cases}$$

which yields

$$\hat{g}(x) = \begin{cases} \int_{-1}^{x} \hat{P}_L(a)\sqrt{x-a}\,da & -1 < x < 0 \\ \int_{-1}^{0} \hat{P}_L(a)\sqrt{x-a}\,da + \int_0^x \hat{P}_R(a)\sqrt{x-a} & 0 < x < 1 \end{cases}$$

$\hat{g}(x)$ is continuous at $x = 0$, and so we cannot compute a non-smooth function analytically with this type of weighted decoder. If we attempt to use a δ pulse for $\hat{P}(a)$, $\hat{P}(a) = \delta(a)\gamma(a)$ then all we arrive at is

$$\hat{g}(x) = \gamma(0)\sqrt{x} = k\sqrt{x}$$

and so we can get the $\sqrt{x}$ function. From the preceding argument however, it should be clear that we can get an arbitrary piecewise smooth continuous function. In fact, one can be constructed rather easily. Consider the two operators

$$\hat{g}_R^+(x) = \int_0^x \hat{P}_R^+(a)\sqrt{x-a} \quad 0 < x < 1 \tag{2}$$

$$\hat{g}_L^-(x) = \int_0^{-x} \hat{P}_L^-(a)\sqrt{-x-a} \quad -1 < x < 0 \tag{3}$$

The first thing to realize is that with these two operators, we can approximate any function that vanishes to first order at $x = 0$. For example, we have

$$\hat{P}_R^+(a) = \frac{2}{\pi}\frac{g_R'(0)}{\sqrt{a}} + \int_0^a \frac{2}{\pi\sqrt{t}} g_R''(a-t)\,dt \tag{4}$$

$$\hat{P}_L^-(a) = \frac{2}{\pi}\frac{g_L'(0)}{\sqrt{a}} + \int_0^a \frac{2}{\pi\sqrt{t}} g_L''(t-a)\,dt \tag{5}$$

Note that $g_R^+(x)$ and $g_L^-(x)$ both equal 0 when $x = 0$ and thus we can compute a piecewise smooth continuous function that is 0 on the switching boundary. To compute a function that is non-zero on the switching boundary, we can merely use the following:

$$\hat{P}^+(a) = \begin{cases} \frac{1}{\pi}\frac{g(0)}{\sqrt{1+a}} & a < 0 \\ \frac{1}{\pi}\frac{g(0)}{\sqrt{1+a}} + \frac{2}{\pi}\frac{g_R'(0)}{\sqrt{a}} + \int_0^a \frac{2}{\pi\sqrt{t}} g_R''(a-t)\,dt & 0 < a < 1 \end{cases} \tag{6}$$

$$\hat{P}^-(a) = \begin{cases} \frac{1}{\pi}\frac{g(0)}{\sqrt{1+a}} & a < 0 \\ \frac{1}{\pi}\frac{g(0)}{\sqrt{1+a}} + \frac{2}{\pi}\frac{g_L'(0)}{\sqrt{a}} + \int_0^a \frac{2}{\pi\sqrt{t}} g_L''(t-a)\,dt & 0 < a < 1 \end{cases} \tag{7}$$

where $g(0) = g_L(0) = g_R(0)$. This allows us to compute an arbitrary piecewise smooth-continuous function.

2 POLYNOMIAL RECURRENCE RELATIONSHIP

In order to be able to approximate any arbitrary function, we will have to use a basis to basis mapping. The first thing to note is the following recurrence relationship

$$\begin{aligned}
A_n(x) &= \int_{-1}^{x} \frac{(1+a)^n}{2\sqrt{1+a}}\sqrt{x-a}\,da \\
&= \int_0^{\sqrt{x+1}} u^{2n}\sqrt{x+1-u^2}\,du \\
&= -\frac{1}{3}u^{2n-1}((x+1)-u^2)^{3/2}\Big|_0^{\sqrt{x+1}} + \int_0^{\sqrt{x+1}} \frac{2n-1}{3}(x+1-u^2)u^{2n-2}\sqrt{x+1-u^2}\,du \\
&= \frac{2n-1}{3}(x+1)\int_0^{\sqrt{x+1}} u^{2(n-1)}\sqrt{x+1-u^2}\,du - \frac{2n-1}{3}\int_0^{\sqrt{x+1}} u^{2n}\sqrt{x+1-u^2}\,du \\
&= \frac{2n-1}{3}(x+1)A_{n-1}(x) - \frac{2n-1}{3}A_n(x) \\
A_n(x) &= (x+1)\left(\frac{2n-1}{2n+2}\right)A_{n-1}(x)
\end{aligned}$$

for $n > 2$ Also note that

$$A_0(x) = \int_{-1}^{x} \frac{1}{2\sqrt{1+a}}\sqrt{x-a}\,da = \frac{\pi}{4}(x+1)$$

and thus if we initialize the recurrence with $B_0(x) = \frac{4}{\pi}A_0(x)$ we obtain the following:

$$
\begin{aligned}
B_0(x) &= (x+1) \\
B_1(x) &= \frac{1}{4}(x+1)^2 \\
B_2(x) &= \frac{1}{8}(x+1)^3 \\
B_n(x) &= \frac{(2n-1)(2n-3)\ldots 5\cdot 3\cdot 1}{2^n(n+1)!}(x+1)^n
\end{aligned}
$$

3 THE EXPECTED ERROR

In this section, we will derive the expected squared error:

$$
\begin{aligned}
E((\hat{g}_{2N}(x)-g(x))^2) &= E((\hat{g}_N^+(x)+\hat{g}_N^-(x)-g(x))^2 && (8) \\
&= E\left(\frac{1}{N}\sum_{j=1}^{N}\gamma^+(a_i^+)f(x-a_i)+\frac{1}{N}\sum_{j=1}^{N}\gamma^-(a_i^-)f(-x-a_i)-g(x)\right)^2 && (9)
\end{aligned}
$$

To simplify things a little bit we have used N neurons in each population (ON/OFF) as opposed to $N/2$ to avoid having to carry around the factors of 2 that come up during the derivation. The final answer can be rescaled to yield the expectation for $\hat{g}_N(x)$ instead of $\hat{g}_{2N}(x)$. where $g(x) = g^+(x) + g^-(x)$. Note that the factor of 2 present in each $\hat{g}_\pm(x)$ in equation (30) of the text can be absorbed into the $\gamma(a_i)$ without loss of generality. Expanding out line (9) yields the following

$$
\begin{aligned}
E((\hat{g}_{2N}(x)-g(x))^2) &= E\left(\frac{1}{N^2}\sum_{i=1}^{N}\gamma^+(a_i^+)^2f(x-a_i)^2+\frac{1}{N^2}\sum_{i=1}^{N}\gamma^-(a_i^-)^2f(-x-a_i)^2+g(x)^2\right) \\
&- 2g(x)E\left(\frac{1}{N}\sum_{i=1}^{N}\gamma^+(a_i^+)f(x-a_i)\right)-2g(x)E\left(\frac{1}{N}\sum_{i=1}^{N}\gamma^-(a_i^-)f(-x-a_i)\right) \\
&+ 2E\left[\left(\frac{1}{N}\sum_{i=1}^{N}\gamma^+(a_i^+)f(x-a_i)\right)\cdot\left(\frac{1}{N}\sum_{i=1}^{N}\gamma^-(a_i^-)f(-x-a_i)\right)\right] \\
&+ E\left(\frac{1}{N^2}\sum_{i\neq j}\gamma^+(a_i^+)\gamma^+(a_j^+)f(x-a_i^+)f(x-a_j^+)\right) \\
&+ E\left(\frac{1}{N^2}\sum_{i\neq j}\gamma^-(a_i^-)\gamma^-(a_j^-)f(-x-a_i^-)f(x-a_j^-)\right)
\end{aligned}
$$

As a_i^+ and a_i^- are drawn from independent identically distributed random variables, then we have the following

$$E\left(\frac{1}{N^2}\sum_{i=1}^{N}\gamma^+(a_i^+)^2 f(x-a_i)^2\right) = \frac{1}{N}\int_{-1}^{x}\gamma^+(a)^2\rho(a)f(x-a)^2\,da \tag{10}$$

$$E\left(\frac{1}{N^2}\sum_{i=1}^{N}\gamma^-(a_i^-)^2 f(-x-a_i)^2\right) = \frac{1}{N}\int_{-1}^{-x}\gamma^-(a)^2\rho(a)f(-x-a)^2\,da \tag{11}$$

$$E\left[\left(\frac{1}{N}\sum_{i=1}^{N}\gamma^+(a_i^+)f(x-a_i)\right)\cdot\left(\frac{1}{N}\sum_{i=1}^{N}\gamma^-(a_i^-)f(-x-a_i)\right)\right] = g^+(x)g^-(x) \tag{12}$$

$$E\left(\frac{1}{N^2}\sum_{i\neq j}\gamma^+(a_i^+)\gamma^+(a_j^+)f(x-a_i^+)f(x-a_j^+)\right) = \left(\frac{N^2-N}{N^2}\right)g^+(x)^2 \tag{13}$$

$$E\left(\frac{1}{N^2}\sum_{i\neq j}\gamma^-(a_i^-)\gamma^-(a_j^-)f(-x-a_i^-)f(x-a_j^-)\right) = \left(\frac{N^2-N}{N^2}\right)g^-(x)^2 \tag{14}$$

putting all these together yields

$$\begin{aligned}
E((\hat{g}_{2N}(x)-g(x))^2) &= \frac{1}{N}\int_{-1}^{x}\gamma^+(a)^2\rho(a)f(x-a)^2\,da + \frac{1}{N}\int_{-1}^{-x}\gamma^-(a)^2\rho(a)f(-x-a)^2\,da \\
&+ g(x)^2 - 2g(x)(g^+(x)+g^-(x)) + 2g^+(x)g^-(x) + \left(\frac{N^2-N}{N^2}\right)g^+(x)^2 \\
&+ \left(\frac{N^2-N}{N^2}\right)g^-(x)^2 \\
&= \frac{1}{N}\left(\int_{-1}^{x}\gamma^+(a)^2\rho(a)f(x-a)^2\,da - g^+(x)^2\right) \\
&+ \frac{1}{N}\left(\int_{-1}^{x}\gamma^+(a)^2\rho(a)f(x-a)^2\,da - g^-(x)^2\right) \\
&+ -g(x)^2 + 2g^+(x)g^-(x) + g^+(x)^2 + g^-(x)^2 \qquad (15)
\end{aligned}$$

where line (15) is identically zero as $g(x) = g^+(x) + g^-(x)$. Thus, we have the following

$$E((\hat{g}_N(x)-g(x))^2) = \frac{2}{N}\int_{-1}^{x}\gamma^+(a)^2\rho(a)f(x-a)^2\,da + \frac{2}{N}\int_{-1}^{-x}\gamma^-(a)^2\rho(a)f(-x-a)^2\,da$$

4 THE ASYMPTOTIC DISTRIBUTION OF $C(\gamma_N)$.

In this section, we will prove the first two moments of $C(\gamma_N)$ have the following asymptotics

$$E(C(\gamma_N)) \leq O(N^{-1}), \quad E(C(\gamma_N)^2) \leq O(N^{-2}) \tag{16}$$

which immediately implies that

$$E((C(\gamma_N) - E(C(\gamma_N)))^2) \leq O(N^{-2})$$

and that as $N \to \infty$, $C(\gamma_N) \to 0$ in a mean-squared. We will perform this proof with $C(\gamma_N)$ for only the population of ON neurons, with a similar result holding for the population of OFF neurons and both populations together.

4.1 The First Moment of $C(\gamma_N)$

Recall that

$$C(\gamma_N) = \int_{-1}^{1} \left(\frac{1}{N} \sum_{i=1}^{N} \gamma(a_i)\sqrt{x - a_i} - g^+(x) \right)^2 dx + \frac{\lambda}{N} \sum_{i=1}^{N} \frac{\gamma(a_i)^2}{N} \tag{17}$$

We will now write down the following

$$e_N(x) = \frac{1}{N} \sum_{i=1}^{N} \gamma(a_i)\sqrt{x - a_i} - g^+(x) \tag{18}$$

It is simple to prove the following:

$$E(e_N(x)) = 0, \quad E(e_N(x)^2) = \frac{1}{N} e(x) \tag{19}$$

where

$$e(x) = \int_{-1}^{x} \gamma^+(a)^2 \rho_a(a) f(x-a)^2 \, da - g^+(x)^2$$

The condition that

$$\int_{-1}^{1} \gamma^+(a)^2 \rho_a(a) < \infty$$

implies that $e(x)$ is bounded if $f(x - a) < F$ for some F:

$$e(x) \leq \int_{-1}^{x} \gamma^+(a) f(x-a)^2 \, da \leq F^2 \int_{-1}^{x} \gamma^+(a)^2 \rho_a(a) \, da \leq F^2 E(\gamma(a_i)^2)$$

Technically this is only true if $f(x - a)$ is bounded, but for the physical reason that neurons have finite firing rates, we never have to consider this case. Now, we can return to the quantity $e_N(x)$, and note that as it is a linear combination of continuous functions in x, it is also bounded. Additionally, as we are on the closed interval $[-1, 1]$, it attains its upper bound at some point (possibly non-unique), x^* such that

$e_N(x) < e_N(x^*)$. Thus,

$$\begin{aligned} C(\gamma_N) &= \int_{-1}^{1} e_N(x)^2\,dx + \frac{\lambda}{N}\sum_{i=1}^{N}\frac{\gamma(a_i)^2}{N} && (20)\\ &\leq 2e_N(x^*)^2 + \frac{\lambda}{N}\sum_{i=1}^{N}\frac{\gamma(a_i)^2}{N} && (21)\\ E(C(\gamma_N)) &\leq 2E(e_N(x^*)^2) + \frac{\lambda}{N}E(\gamma(a_i)^2) = N^{-1}(2F^2+\lambda)E(\gamma(a_i)^2) && (22) \end{aligned}$$

4.2 The Second Moment of $C(\gamma_N)$

The second moment is be computed by taking the expectation of $C(\gamma_N)^2$. Note the following:

$$\begin{aligned} C(\gamma_N)^2 &= \left(\int_{-1}^{1} e_N(x)^2\,dx + \frac{\lambda}{N}\sum_{i=1}^{N}\frac{\gamma(a_i)^2}{N}\right) && (23)\\ &= \left[\int_{-1}^{1} e_N(x)^2\,dx\right]^2 + 2\int_{-1}^{1} e_N(x)\,dx\left(\frac{\lambda}{N}\sum_{i=1}^{N}\frac{\gamma(a_i)^2}{N}\right) + \frac{\lambda^2}{N^4}\left(\sum_{i=1}^{N}\gamma(a_i)^4 + \sum_{i\neq j}\gamma(a_i)^2\gamma(a_j)^2\right)\\ &\leq 4e_N(x^*)^4 + 4e_N(x^*)^2\frac{\lambda}{N}\left(\sum_{i=1}^{N}\frac{\gamma(a_i)^2}{N}\right) + \frac{\lambda^2}{N^4}\left(\sum_{i=1}^{N}\gamma(a_i)^4 + \sum_{i\neq j}\gamma(a_i)^2\gamma(a_j)^2\right) && (24)\\ &\leq 4e_N(x^*)^4 + \frac{4\lambda}{N}e_N(x^*)^2\gamma^2 + \frac{\lambda^2}{N^4}\left(\sum_{i=1}^{N}\gamma(a_i)^4 + \sum_{i\neq j}\gamma(a_i)^2\gamma(a_j)^2\right) && (25) \end{aligned}$$

where $\gamma(a) < \gamma, a \in [-1, 1]$. Taking the expectation yields

$$\begin{aligned} E(C(\gamma_N)^2) &\leq 4E(e_N(x^*)^4) + \frac{4\lambda\gamma^2}{N}E(e_N(x^*)^2) + \frac{\lambda^2}{N^3}\left(E(\gamma(a_i)^4) + \frac{N-1}{N}E(\gamma(a_i)^2)^2\right)\\ &\leq 4E(e_N(x^*)^4) + \frac{4F^2\lambda\gamma^2}{N^2}E(\gamma(a_i)^2) + \frac{\lambda^2}{N^3}\left(E(\gamma(a_i)^4) + \frac{N-1}{N}E(\gamma(a_i)^2)^2\right) && (26) \end{aligned}$$

Now, as $e_N(x^*)$ is a normally distributed random variable asymptotically (by the central limit theorem) with mean 0 and $\sigma^2 = e(x^*)/N$. We have

$$E(e_N(x^*)^4) \sim 3E(e_N(x^*)^2)^2 \leq 3\left(\frac{F^2}{N}E(\gamma(a_i)^2)\right)^2 \quad (27)$$

based on the unique property of the normal distribution that all higher order moments are defined by the first two moments. Finally we have

$$\begin{aligned} E(C(\gamma_N)^2) &\leq \frac{12F^4}{N^2}E(\gamma(a_i)^2)^2 + \frac{4F^2\lambda\gamma^2}{N^2}E(\gamma(a_i)^2) + \frac{\lambda^2}{N^3}\left(E(\gamma(a_i)^4) + \frac{N-1}{N}E(\gamma(a_i)^2)^2\right) \\ &\sim O(N^{-2}) \end{aligned} \tag{28}$$

An important thing to note is that the assumption on $\gamma(a)$ being bounded immediately yields the existence of $E(\gamma(a_i)^k)$ for all k, while we only need the existence of the moment for $k = 4$ at most in order to prove that $E(C(\gamma_N)^2) \leq O(N^{-2})$, and thus there is some room to loosen the conditions on this proof.

5 NON-UNIQUENESS FOR MULTI-VARIABLE FUNCTION REPRESENTATION

Consider the two-dimensional function $g(x, y) = xy$. With a two-dimensional polar coordinate system, we will assume all the decoding vectors are evenly distributed as follows:

$$\rho_\theta(\theta) = \frac{1}{4}\delta\left(\theta - \frac{\pi}{4}\right) + \frac{1}{4}\delta\left(\theta - \frac{3\pi}{4}\right) + \frac{1}{4}\delta\left(\theta - \frac{5\pi}{4}\right) + \frac{1}{4}\delta\left(\theta - \frac{7\pi}{4}\right)$$

and thus, $\hat{P}(\theta)$ must also be δ valued:

$$\hat{P}_\theta(\theta) = \frac{A}{4}\delta\left(\theta - \frac{\pi}{4}\right) + \frac{B}{4}\delta\left(\theta - \frac{3\pi}{4}\right) + \frac{C}{4}\delta\left(\theta - \frac{5\pi}{4}\right) + \frac{D}{4}\delta\left(\theta - \frac{7\pi}{4}\right)$$

where $A, B, C,$ and D are yet to be determined and so we have the following:

$$\begin{aligned}
&\int_0^{2\pi} \int_{-1}^{\cos(\theta)x+\sin(\theta)y} \hat{P}_a(a)\hat{P}_\theta(\theta)\sqrt{\cos(\theta)x + \sin(\theta)y - a}\, da\, d\theta \\
&= \frac{A}{4}\int_{-1}^{x/\sqrt{2}+y/\sqrt{2}+1} \hat{P}_a(a)\sqrt{\frac{x}{\sqrt{2}} + \frac{y}{\sqrt{2}} - a}\, da \\
&+ \frac{B}{4}\int_{-1}^{-x/\sqrt{2}+y/\sqrt{2}+1} \hat{P}_a(a)\sqrt{-\frac{x}{\sqrt{2}} + \frac{y}{\sqrt{2}} - a}\, da \\
&+ \frac{C}{4}\int_{-1}^{-x/\sqrt{2}-y/\sqrt{2}+1} \hat{P}_a(a)\sqrt{-\frac{x}{\sqrt{2}} - \frac{y}{\sqrt{2}} - a}\, da \\
&+ \frac{D}{4}\int_{-1}^{x/\sqrt{2}-y/\sqrt{2}+1} \hat{P}_a(a)\sqrt{\frac{x}{\sqrt{2}} - \frac{y}{\sqrt{2}} - a}\, da
\end{aligned} \tag{29}$$

Now, from our previous work, we know that if we take

$$\hat{P}_a(a) = \frac{8}{\pi}\sqrt{a+1},$$

then we have the following:

$$\begin{aligned}
\hat{g}(x, y) &= \frac{A}{4}\left(\frac{x}{\sqrt{2}} + \frac{y}{\sqrt{2}} + 1\right)^2 + \frac{B}{4}\left(-\frac{x}{\sqrt{2}} + \frac{y}{\sqrt{2}} + 1\right)^2 + \frac{C}{4}\left(-\frac{x}{\sqrt{2}} - \frac{y}{\sqrt{2}} + 1\right)^2 \\
&+ \frac{D}{4}\left(\frac{x}{\sqrt{2}} - \frac{y}{\sqrt{2}} + 1\right)^2 \\
&= \frac{x^2}{8}(A + B + C + D) + \frac{y^2}{8}(A + B + C + D) + \frac{xy}{4}(A - B + C - D) \\
&+ \frac{\sqrt{2}x}{4}(A - B - C + D) + \frac{\sqrt{2}y}{4}(A + B - C - D) + \frac{1}{4}(A + B + C + D)
\end{aligned}$$

now, while we have 6 terms in the polynomial, three of the coefficients that are unnecessary are identical. This yields the four linear equations:

$$\begin{aligned} A+B+C+D &= 0 \\ A-B-C+D &= 0 \\ A+B-C-D &= 0 \\ A-B+C-D &= 4 \end{aligned}$$

which yields $\hat{g}(x,y) = xy$ when $A = C, B = D, A = -B = 1$. And thus, the resulting scale invariant decoders are given by

$$\gamma(a) = \frac{8}{\pi}\sqrt{a+1} \tag{30}$$

for all 4 discrete sub-populations. This is one particular solution, however consider equation (29) but instead let us use a different $\hat{P}$ for all the discrete subpopulations. defining $z_1 = (x+y)/\sqrt{2}4$ and $z_2 = (x-y)/\sqrt{2}$, then we have $g(x,y) = h(z_1, z_2) = z_1^2 - z_2^2$ and

$$\hat{h}(z_1, z_2) = L^+_{z_1}(\hat{P}^+_{z_1}(a)) + L^-_{z_1}(\hat{P}^-_{z_1}(a)) + L^+_{z_2}(\hat{P}^+_{z_2}(a)) + L^-_{z_2}(\hat{P}^-_{z_2}(a)) \tag{31}$$

where the subscripts denote the independent variable in the function range. Given our previous discussion of non-uniqueness in the single variable case, it should be clear that we can use functions $\epsilon(z_1)$ and $\epsilon(z_2)$ where $\epsilon(z_1)$ is in the function range of both $L^{\pm}_{z_1}$ with an identical condition holding for $\epsilon(z_2)$ to create non-unique $\hat{P}^{\pm}$.
